# Nectin-4 promotes osteosarcoma progression and metastasis through activating PI3K/AKT/NF-κB signaling by down-regulation of miR-520c-3p

**DOI:** 10.1186/s12935-022-02669-w

**Published:** 2022-08-11

**Authors:** Yongheng Liu, Guanghao Li, Yan Zhang, Lili Li, Yanting Zhang, Xiaoyu Huang, Xianfu Wei, Peng Zhou, Ming Liu, Gang zhao, Jinyan Feng, Guowen Wang

**Affiliations:** 1 Department of Bone and Soft Tissue Tumors, Tianjin Medical University Cancer Institute and Hospital, National Clinical Research Center for Cancer, Huanhu Xi Road, Tiyuan Bei, Hexi District, Tianjin, 300060 People’s Republic of China; 2 Key Laboratory of Cancer Prevention and Therapy, Tianjin, China; 3 Tianjin’s Clinical Research Center for Cancer, Tianjin, China; 4 Department of Orthopedics, Affiliated Hospital of Chifeng University, Chifeng, Inner Mongolia China; 5 Department of Pathology, Tianjin Medical University Cancer Institute and Hospital, National Clinical Research Center for Cancer, Tianjin, China

**Keywords:** Nectin-4, Osteosarcoma, Metastasis, PI3K/AKT/NF-κB, miR-520c-3p

## Abstract

**Purpose:**

Nectin-4 is specifically up-regulated in various tumors, exert crucial effects on tumor occurrence and development. Nevertheless, the role and molecular mechanism of Nectin-4 in osteosarcoma (OS) are rarely studied.

**Methods:**

The expression of Nectin-4 and its relationship with clinical characteristics of OS were investigated using OS clinical tissues, tissue microarrays, TCGA, and GEO databases. Moreover, the effect of Nectin-4 on cell growth and mobility was detected by CCK-8, colony formation, transwell, and wound-healing assays. The RT-qPCR, Western blotting, and luciferase reporter assays were performed to explore molecular mechanisms through which Nectin-4 mediates the expression of miR-520c-3p, thus modulating PI3K/AKT/NF-κB signaling. In vivo mice models constructed by subcutaneous transplantation and tail vein injection were used to validate the functional roles of Nectin-4 and miR-520c-3p.

**Results:**

Nectin-4 displayed a higher expression in OS tumor tissues compared with normal tissues, and its overexpression was positively associated with tumor stage and metastasis in OS patients. Functionally, Nectin-4 enhanced OS cells growth and mobility in vitro. Mechanistically, Nectin-4 down-regulated the levels of miR-520c-3p that directly targeted AKT-1 and P65, thus leading to the stimulation of PI3K/AKT/NF-κB signaling. In addition, the expression of miR-520c-3p was apparently lower in OS tissues than in normal tissues, and its low expression was significantly related to tumor metastasis. Furthermore, ectopic expression of miR-520c-3p markedly blocked the effect of Nectin-4 on OS cell growth and mobility. Knockdown of Nectin-4 could suppress the tumorigenesis and metastasis in vivo, which could be remarkably reversed by miR-520c-3p silencing.

**Conclusions:**

Nectin-4 as an oncogene can promote OS progression and metastasis by activating PI3K/AKT/NF-κB signaling via down-regulation of miR-520c-3p, which could represent a novel avenue for identifying a potential therapeutic target for improving patient outcomes.

**Supplementary Information:**

The online version contains supplementary material available at 10.1186/s12935-022-02669-w.

## Introduction

Osteosarcoma (OS) is currently considered as one of the most common malignant bone tumors, which is usually found in young individuals aged 10 ~ 30 years old and has a high propensity for tumor recurrence and early metastasis [1, 2]. With the current multi-disciplinary therapy, the 5-year survival rate in OS patients has been found to increase by 50 ~ 60% compared with surgical treatment alone [3, 4]. Unfortunately, numerous patients still die due to the fast disease progression and intense tumor invasion [5]. Lung metastasis is one of the most common reasons for OS-related deaths, as patients with lung metastasis are at greater risk of a shorter 5-year event-free survival, which drops from 60 to 25% [6–8]. Therefore, gaining more in-depth insight into molecular mechanisms of OS and identifying novel molecular biomarkers and therapeutic targets is of great significance [9–12].

Numerous studies have confirmed that the disorder of cell–cell adhesion significantly influences tumor occurrence and development [13, 14]. The Nectin cell adhesion molecule (Nectin) family comprises Nectin-1 ~ 4, which belong to immunoglobulin-semblable transmembrane proteins involved in the Ca^2+^ independent adherens junctions (AJs) of cell–cell and have a vital role in enhancing cellular viability and movement ability [15–17]. However, numerous studies revealed that Nectin-4 is specifically up-regulated in various cancers, including breast, lung, ovarian, gastric, and pancreatic cancer, and its expression is closely related to tumor oncogenesis and worse prognosis of the patients [18–23]. In addition, it has also been reported that Nectin-4 could activate the phosphoinositide 3-kinase (PI3K)/protein kinase B (AKT) pathway and is related to tumor proliferation, metastasis, and angiogenesis in the gallbladder and gastric cancer [24, 25]. Nevertheless, little is known about the functional role of Nectin-4 protein in OS.

MicroRNAs (miRNAs) are a major class of short (18 ~ 25 nts), non-coding single-stranded RNAs that can directly affect gene expression and, in turn, regulate biological functionality [26]. These miRNAs target and bind to the mRNAs′ specific 3′-untranslated region (3′-UTR) and then degrade the mRNA at the posttranscriptional level [27, 28]. An increasing number of studies revealed that miRNAs have a vital role in the tumor growth, progression, and metastasis of OS by modulating target mRNA [29–31]. For instance, Zhong et al. [29] reported that high expression of miR-1270 had a strong positive correlation with poor prognosis OS patients. Moreover, Zhang and colleagues [30] reported that miR-493-5p could significantly restrain tumorigenesis and metastasis of OS. Recently, numerous studies have confirmed that miR-520c-3p is strongly associated with the occurrence and progression of numerous tumors [32–34]. However, the functional roles of miR-520c-3p in OS remain largely unknown.

In the present study, we confirmed that Nectin-4 protein served as a crucial oncogene regulating OS cells proliferation, migration, invasion, and metastasis both in vitro and in vivo. Moreover, we also identified the molecular mechanisms through which Nectin-4 activates PI3K/AKT/NF-κB signaling mediated by modulating miR-520c-3p.

## Materials and methods

### Tissue samples

For purpose of detecting the Nectin-4 expression, a total number of 29 OS and 8 normal tissue samples (containing 8 pairs of samples) were obtained from OS patients who had not accepted chemotherapy and/or radiotherapy before surgical resection in Tianjin Medical University Cancer Institute and Hospital. Informed consent to participate in research was obtained. All collected tissues were stored at liquid nitrogen. Additionally, tissue microarrays (TMAs) used for immunohistochemical (IHC) staining analysis were composed of 71 samples including 1 normal bone tissue and 70 OS tissues, sampled from 2 patients with stage IA, 31 patients with stage IIA, 31 patients with stage IIB, and 6 patients with stage IVB (Bioaitech, cat.no. L714901; Xi′an, China). This study was authorized and supervised by the Ethics Committee of the Tianjin Medical University Cancer Institute and Hospital.

### Conducting subcutaneous xenograft and lung metastatic mice models

The BALB/c-nude mice (female, 4 ~ 5 weeks old) were purchased from Beijing SPF Biotechnology Co., Ltd. All animal operations and procedures were authorized by the Animal Care Committee of the Tianjin Medical University Cancer Institute and Hospital, China.

To detect the functional role of Necitn-4 in vivo, wo conducted subcutaneous xenograft and lung metastatic mice models. For a subcutaneous xenograft model, the nude mice were randomly divided in 2 groups: the shCtrl group and the shNectin-4#2 group (5 mice/group). For details, 5 × 10^6^ (resuspended in 100 μl saline solution) stable shCtrl or shNectin-4#2 143B cells were subcutaneously injected into the flank of BALB/c-nude mice. In addition, the tumor growth size was observed and recorded every 3 days using standard calipers. Finally, mice were euthanized 24 days later after subcutaneous injection, after which the tumors were harvested, photographed, and weighted. Sections were subjected to HE staining. IHC and Western blotting was performed to measure the expression of Nectin-4, ZO-1, Vimentin, and Ki-67 in tumor tissues. For the lung metastatic model, 1 × 10^6^ stable shCtrl or shNectin-4 143B cells (re-suspend with 100 μl saline solution) were injected into the nude mice by lateral tail vein (5 mice/group). Three weeks after implantation, those mice were euthanized, and lung tissues were removed.

To detect the effect of miR-520c-3p in vivo, 5 × 10^6^ stable shNectin-4 143B cells transfected with 50 nM miR-520c-3p or NC inhibitor were subcutaneously injected into the nude mice (6 mice/group), after which 5 µg of miR-520c-3p/NC inhibitor (100 μl volume) was injected into the tumor on the 9, 12, and 15 days after cell inoculation [28]. The follow-up tests were carried out as described above.

### Statistical analysis

Data conforming to normal distribution were displayed with mean ± SD. The relevance between Nectin-4 and clinicopathological indicators was evaluated by the χ2 test or Chi-square analysis. The correlation between Nectin-4 and miR-520c-3p was evaluated by Spearman’s analysis. Furthermore, statistical comparison and plotting of those experimental data were carried out by Student’s *t*-test, the Wilcoxon signed-rank test, the ANOVA analysis, and the Mann–Whitney test, which was performed with SPSS software version 22.0 (IBM SPSS, Armonk, NY, USA) and GraphPad Prism 8 (GraphPad Software, Inc., La Jolla, CA, USA). A *P*-value of < 0.05 was regarded to be statistically significant. Each assay was repeated at least three times.

## Results

### The Nectin‑4 expression and clinical significance in OS

**Fig. 1:**
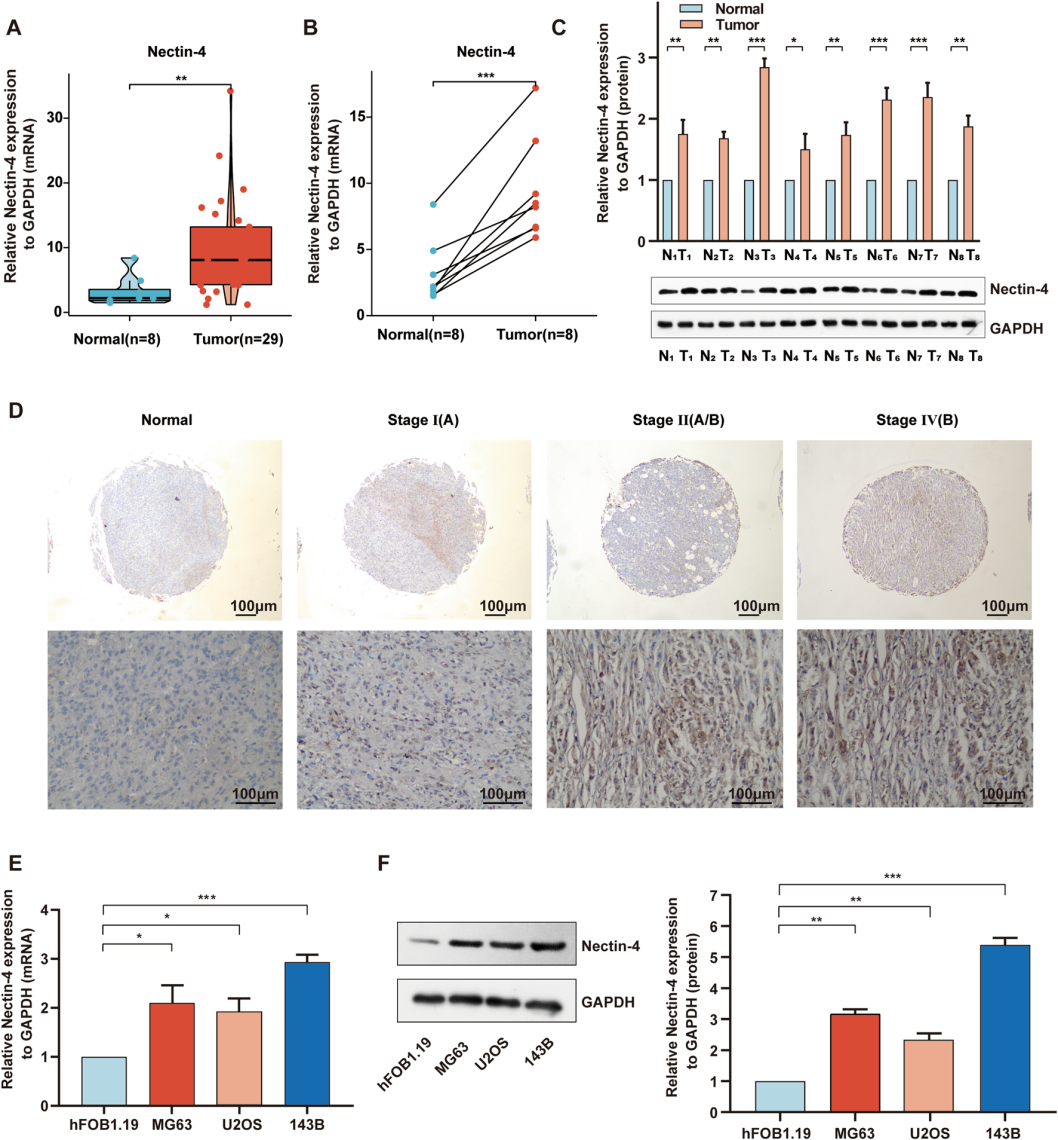
The Nectin‑4 expression and clinical significance in OS. **A** The expression of Nectin-4 in OS (n = 29) and adjacent normal tissues (n = 8) by using RT-qPCR analysis. **B**, **C** The expression of Nectin-4 in the 8 paired samples of OS and adjacent normal tissues at mRNA and protein levels. **D** Immunohistochemical (IHC) stain analysis of Nectin-4 expression in OS specimens and normal tissues on tissue microarrays (scale bars 100 μm, magnifications of 200×). **E**, **F** The basal expression levels of Nectin-4 in OS cell lines (MG63, U2OS, and 143B) and osteoblastic cell line (hFOB1.19) by using RT-qPCR and Western blotting. Each assay was repeated at least three times. ns, no significance; *P < 0.05; **P < 0.01; ***P < 0.001

Nectin 1 ~ 3 is commonly enriched in normal adult tissues, while Nectin-4 was found to be specifically up-regulated in various tumors, having a significant role in tumor occurrence and development [15–21]. Nevertheless, little is known about the role of Nectin-4 protein in OS. Firstly, we wanted to monitor the expression levels of Nectin-4 in OS tissues and adjacent normal tissues. By using RT-qPCR, we found that Nectin-4 expression was markedly increased in OS tissues compared to normal tissues (Fig. 1A). Similarly, the significant difference in expression of Nectin-4 was also observed in 8 paired samples of OS and adjacent normal tissues both at mRNA and protein levels (Fig. 1B and C).

In order to further investigate the expression difference of Nectin-4 between OS tissues and normal tissues and the association between Nectin-4 and clinical features, the related gene expression profiles, and clinical data were downloaded from TARGET, GTEx, and GEO databases. Interestingly, the mRNA level of Nectin-4 displayed obvious overexpression in OS tissues using the TARGET database compared with the normal muscle tissue obtained from GTEx database (Additional file 1: Fig. S1A). Furthermore, to evaluate the association between the gene expression of Nectin-4 and clinical features in OS, we re-assayed the data acquired from the GSE21257. The mean value of Nectin-4 mRNA expression was regarded as the threshold, after which the patients were divided into two cohorts (high expression cohort and low expression cohort). As shown in Table 1, the Nectin-4 high expression was strongly associated with tumor metastasis (*P* < 0.05). Strikingly, we found that more patients in the high expression group (16/19, 84.2%) exhibited tumor metastasis compared with the low expression group (18/34, 52.9%), which implies that Nectin-4 might regulate the tumor progression in OS.

**Table 1:** Association between Nectin-4 expression and clinicopathologic features in GSE21257

Variable		No	Nectin-4 expression		x^2^	P-value
			High	Low		
Gender					0.235	0.625
	Male	34	13 (24.53%)	21 (39.62%)		
	Female	19	6 (11.32%)	13 (24.53%)		
Age					0.051	0.821
	≥ 18.7Y	13	5 (9.43%)	8 (15.09%)		
	< 18.7Y	40	14 (26.42%)	26 (49.06%)		
Grade					8.958	0.176
	1	13	7 (13.21%)	6 (11.32%)		
	2	16	5 (9.43%)	11 (20.75%)		
	3	13	2 (3.77%)	11 (20.75%)		
	4	5	3 (5.66%)	2 (3.77%)		
Metastasis					5.182	0.036*
	Yes	34	16 (30.19%)	18 (33.96%)		
	No	19	3 (5.66%)	16 (30.19%)		

Using Chi-square test or Fisher’s exact test

*P < 0.05 was considered statistically significant

To determine the differential expression of Nectin-4 in diverse histologic stages of OS, Nectin-4 IHC was performed on an OS TMA. Overall, 48/70 (68.6%) tumors expressed Nectin-4, with 30/48 (62.5%) tumors showing high-level expression and 18/48 (37.5%) low level. High expression of Nectin-4 was most commonly found in stage IVB OS tissues (66.7%, 4/6), followed by stage IIB (51.6%, 16/31), stage IIA (32.3%, 10/31), and stage IA OS tissues (0%, 0/2), which indicates that high expression of Nectin-4 is closely correlated with higher OS stages (Fig. 1D and Additional file 1: Fig. S1B).

Next, three diverse OS cell lines (MG63, U2OS, and 143B) and one osteoblastic cell line (hFOB1.19) were assessed for basal expression levels of Nectin-4 by RT-qPCR and Western blotting. The results demonstrated that Nectin-4 expression in MG63, U2OS, and 143B cell lines was significantly higher than that in hFOB1.19 cell lines (Fig. 1E and F). The highest up-regulation of Nectin-4 was found in 143B cells, followed by MG63 and U2OS cell lines. Therefore, the 143B cell line was selected for Nectin-4 knockdown, and MG63 and U2OS cell lines were selected for Nectin-4 overexpression. Accordingly, we concluded that Nectin-4 is highly expressed in clinical OS tissues and OS cells.

### Nectin-4 promotes OS cells proliferation

**Fig. 2:**
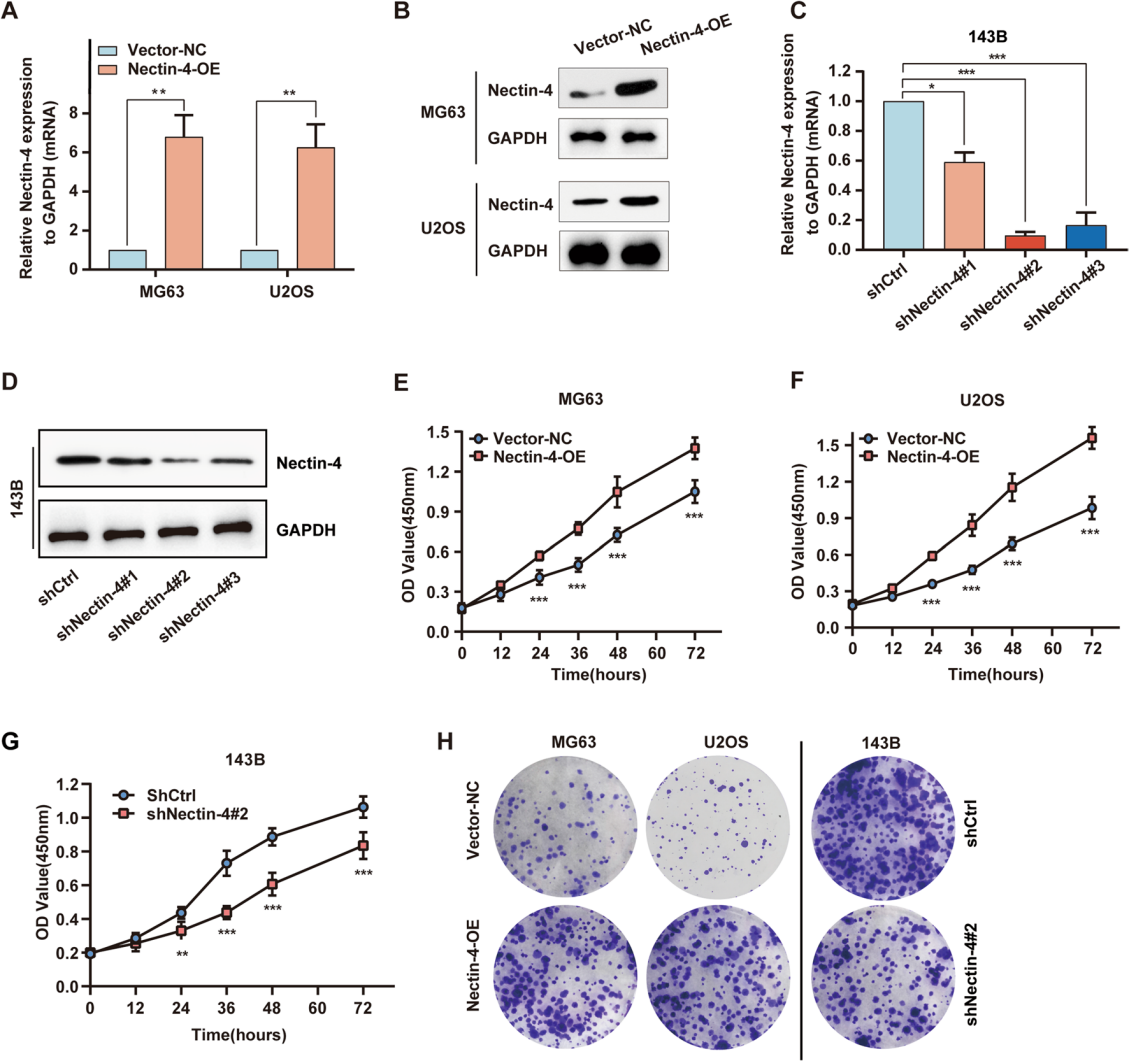
The effect of Nectin-4 on human OS cells proliferation. **A**, **B** The expression of Nectin-4 in MG63 and U2OS cells infected with vector negative control (Vector-NC) or Nectin-4 overexpression (Nectin-4-OE) lentivirus verified by RT-qPCR analysis and Western blotting assay, respectively. **C**, **D** The effectiveness of shNectin-4#1, #2, and #3 at mRNA level in 143B cells verified by RT-qPCR and Western blotting, respectively. **E**, **F** The cell proliferation curve of MG63 and U2OS cells infected with Vector-NC and Nectin-4-OE lentivirus via CCK-8 assay. **G** The cell proliferation curve of 143B cells in shCtrl group and shNectin-4#2 group using CCK-8 assay. **H** Colony formation of MG63, U2OS cells (infected with Vector-NC or Nectin-4-OE lentivirus), and 143B cells (infected with shCtrl or shNectin-4#2 lentivirus). Each assay was repeated at least three times. ns, no significance; *P < 0.05; **P < 0.01; ***P < 0.001

To discuss whether Nectin-4 had an influence on OS cell viability, we firstly constructed and sorted out the OS cell lines with stable Nectin-4 overexpression (Nectin-4-OE) or knockdown by means of lentivirus infection. As shown in Fig. 2A, B and Additional file 2: Fig. S2A, our results confirmed that Nectin-4 was successfully over-expressed in the Nectin-4-OE group of U2OS and MG63 cells compared with vector negative control (Vector-NC) group cells at both mRNA and protein levels. Besides, to further validate the functional roles of Nectin-4 in OS cells, we constructed the stable Nectin-4 knockdown OS cell line. As shown in Fig. 2C, using RT-qPCR, Nectin-4 was significantly down-regulated at mRNA level in 143B cells infected with Lenti-shRNAs, where shNectin-4#2 showed the most potently down-regulated effect compared with shNectin-4#1 and shNectin-4#3. Moreover, the result was validated at protein levels by using Western blotting assays (Fig. 2D and Additional file 2: Fig. S2B). Thus, shNectin-4#2 cells were used for the following investigation.

Next, we investigated the function of Nectin-4 on cell proliferation by using CCK-8 and colony formation assays. As shown in Fig. 2E and F, in comparison with the Vector-NC groups, up-regulation of Nectin-4 remarkably enhanced cell proliferation in both U2OS and MG63 cell lines at different time points (24, 36, 48, and 72 h) (all *P* < 0.001). Moreover, we detected the effect of down-regulated Nectin-4 on OS cell viability. As shown in Fig. 2G, the proliferation rates of shNectin-4#2 group cells were remarkably lower than that of shCtrl group cells at different time points (24, 36, 48, and 72 h) in 143B cell line (*P* < 0.01, respectively). In addition, we also found that up-regulation of Nectin-4 could significantly facilitate colony formation both in MG63 and U2OS cell lines compared with the Vector-NC groups, suggesting that overexpression of Nectin-4 could markedly promote the OS cell proliferation (Fig. 2H and Additional file 2: Fig. S2C; both in MG63 and U2OS, *P* < 0.01). Inversely, down-regulation of Nectin-4 noticeably restrained colony formation in 143B cells compared with the shCtrl group (Fig. 2H and Additional file 2: Fig. S2C, *P* < 0.001). In conclusion, the above results indicated that Nectin-4 could significantly modulate the human OS cells proliferation.

### Nectin-4 enhances human OS cells migration and invasion in vitro

**Fig. 3:**
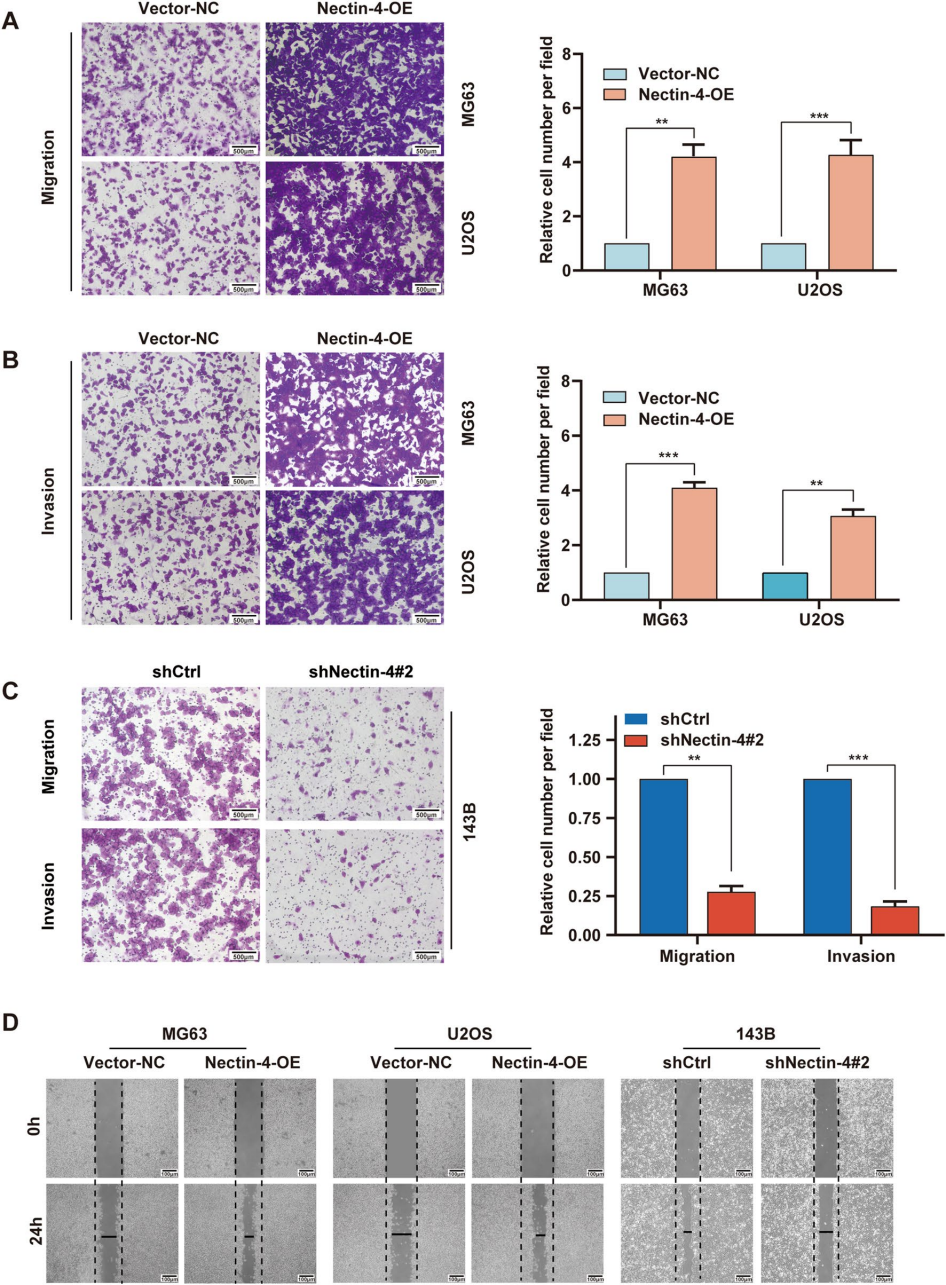
The function of Nectin-4 on human OS cells migration and invasion. **A** The migration ability of the MG63 and U2OS cells with lenti-Nectin-4 or lenti-Vector-NC infection assessed using transwell assay without Matrigel (scale bars 500 μm, magnifications of 100 ×). **B** The invasion ability of MG63 and U2OS cells in Vector-NC group and Nectin-4-OE group via transwell assay with Matrigel (scale bars 500 μm, magnifications of 100×). **C** The migration and invasion abilities of the 143B cells in shCtrl group and shNectin-4#2 group via transwell assay with or without Matrigel (scale bars 500 μm, magnifications of 100×). **D** A wound healing assay was performed to measure the migration capacity of the MG63, U2OS cells (in Nectin-4-OE group and Vector-NC group), and 143B (in shNectin-4#2 group and shCtrl group), respectively (scale bars 100 μm, magnifications of 40×). Each assay was repeated at least three times. ns, no significance; *P < 0.05, **P < 0.01, ***P < 0.001

As mentioned above, a close connection between Nectin-4 up-regulation and tumor metastases was confirmed in the GSE21257. To further investigate the effect of Nectin-4 on cell metastasis, we first adopted the transwell assays to observe whether the overexpression of Nectin-4 could enhance the migratory and invasive capacity of MG63 and U2OS cells. Transwell assay without Matrigel showed more migrated cells tinted with dark blue in the Nectin-4-OE group cells than in the Vector-NC groups (Fig. 3A; in MG63, P < 0.01 and in U2OS, P < 0.001). Similarly, using transwell assay with Matrigel, we confirmed that the number of invasive cells in Nectin-4-OE groups was more than twice those in the control groups (Fig. 3B; in MG63, *P* < 0.001 and in U2OS, *P* < 0.01), implying that Nectin-4 could enhance the OS cell movement ability. Furthermore, we also detected the migration and invasion ability of shNectin-4#2 143B cell lines via transwell assays. As shown in Fig. 3C, knockdown of Nectin-4 remarkably restrained the movement activity of 143B cells; migrated cells and invaded cells were markedly reduced compared with shCtrl cells (*P* < 0.01 and *P* < 0.001, respectively).

In addition, the wound-healing assay was also carried out to validate the function of the Nectin-4 up-regulation on the migration ability in OS cell lines. As demonstrated in Fig. 3D and Additional file 3: Fig. S3, the wound width of the Nectin-4-OE group was remarkably shortened than that of the Vector-NC group (in MG63, *P* < 0.01 and in U2OS, *P* < 0.001), which confirmed that up-regulation of Nectin-4 could remarkably heighten the migratory capacity of the MG63 and U2OS cells. Taken together, up-regulation of Nectin-4 promoted the cell movement activity in human OS cell lines.

Moreover, we further observed the influence of Nectin-4 down-regulation on the migration activity in 143B cells. Compared with shCtrl group, shNectin-4#2 group cells presented a much broader wound width (Fig. 3D and Additional file 3: Fig. S3, *P* < 0.001). Shortly, these results implied that Nectin-4 had important roles in modulating the human OS cell proliferation and movement activities in vitro.

### Nectin-4 modulates epithelial-mesenchymal transition (EMT) via activating PI3K/AKT/NF-κB signal pathway

**Fig. 4:**
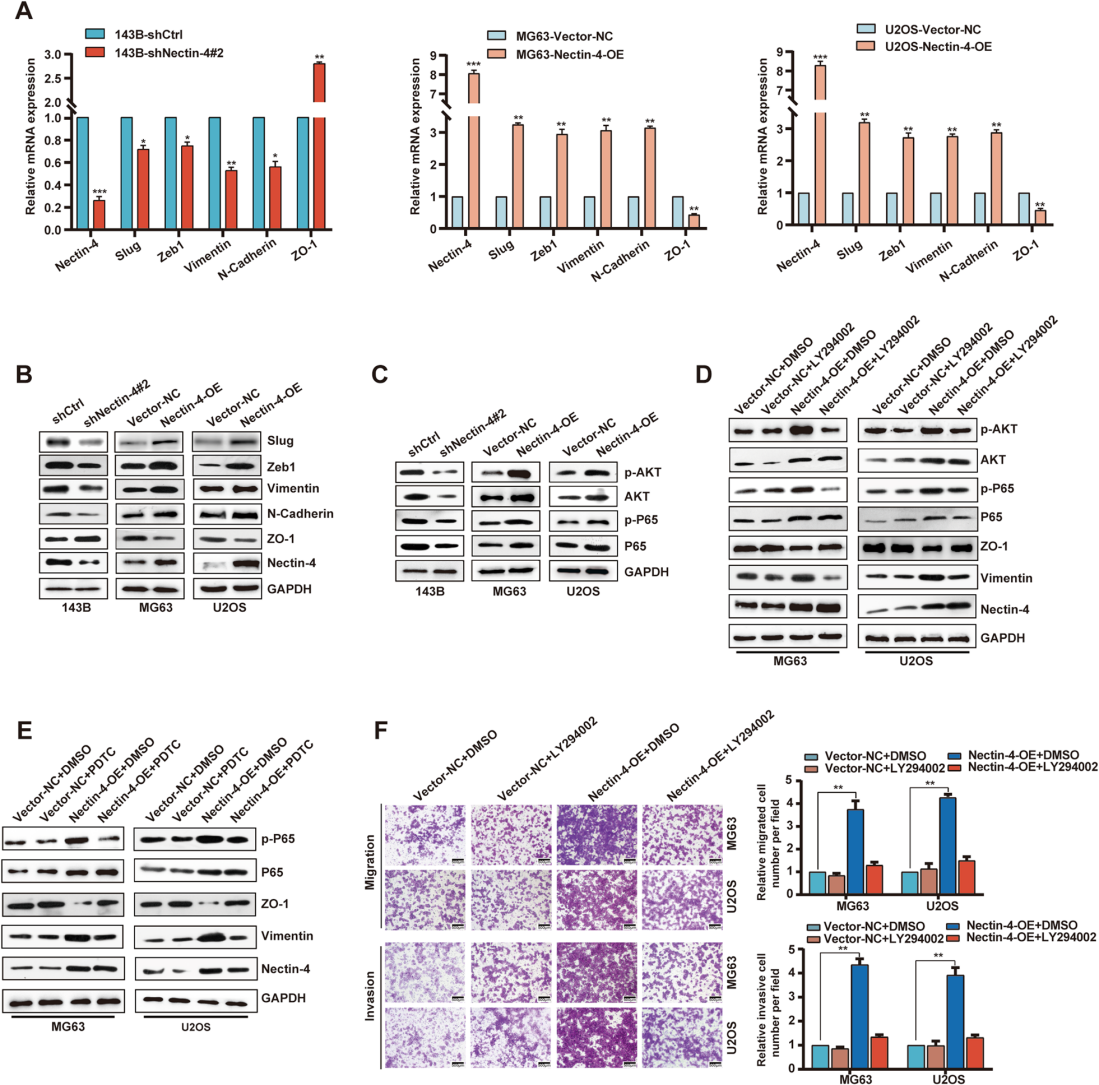
Nectin-4 modulates EMT and migration potency via PI3K/AKT/NF-κB signal pathway. **A** The influences of Nectin-4 on the expression of EMT-related markers in 143B, MG63, and U2OS cell lines (infected with Nectin-4-OE, Vector-NC, shCtrl, and shNectin-4#2 Lentivirus, respectively) by RT-qPCR. **B** The effects of Nectin-4 overexpression or knockdown on the levels of EMT-related markers in cells treated as above by Western blotting. **C** The effects of Nectin-4 overexpression or knockdown on the levels of PI3K/AKT/NF-κB pathway-related markers in cells treated as above by Western blotting. **D** The protein expression levels of EMT-related and PI3K/AKT pathway-related markers in Nectin-4-OE U2OS and MG63 cell lines treated with DMSO and LY294002 (PI3K inhibitor). **E** The protein expression levels of EMT-related and PI3K/AKT pathway-related markers in Nectin-4-OE U2OS and MG63 cell lines treated with DMSO and PDTC (NF-κB inhibitor). **F** The migration and invasion capacity in Nectin-4-OE U2OS and MG63 cell lines treated with DMSO and LY294002 (scale bars 500 μm, magnifications of 200 ×). The concentration of both LY294002 and PDTC was 10 mM, and dissolved in DMSO. Each assay was repeated for at least three times. ns, no significance; *P < 0.05, **P < 0.01, ***P < 0.001

EMT is a classical progress in tumor cell metastasis. We discovered that Nectin-4 has an important role in promoting OS cell movement activities. To further investigate how Nectin-4 could promote OS metastasis, we evaluated the expression changes of typical EMT markers in OS cell lines both at mRNA and protein levels. As shown in Fig. 4A, B, and Additional file 4: Fig. S4A, up-regulation of Nectin-4 led to a reduction of ZO-1 expression, an epithelial marker, and notably elevated the expression of mesenchymal markers including Vimentin and N-Cadherin in U2OS and MG63 cells using RT-qPCR and Western blotting analysis, respectively. Furthermore, we also detected the upstream transcriptional regulators, including Zeb1 and Slug of the EMT signaling pathway. As shown in Fig. 4A, B, and Additional file 4: Fig. S4A, the expression of Zeb1 and Slug was much higher in the group of Nectin-4-OE cells compared to Vector-NC group cells. Additionally, the changes in EMT-related molecule markers were also observed in 143B cells infected with lenti-shNectin-4#2. In contrast with Nectin-4 up-regulation, knockdown of Nectin-4 obviously down-regulated the expression of Vimentin and N-Cadherin, and enhanced the ZO-1 expression (Fig. 4A, B, and Additional file 4: Fig. S4A). Similarly, EMT-related transcription factors (Zeb1 and Slug) also showed the same trend with mesenchymal markers, which were down-regulated when Nectin-4 was depleted (Fig. 4A, B and Additional file 4: Fig. S4A). All these results together confirmed that Necin-4 exerted a crucial influence on regulating EMT.

Numerous studies confirmed that Nectin-4 could facilitate tumor growth, angiogenesis, and metastasis through phosphoinositide 3-kinase/protein kinase B (PI3K/AKT) signaling cascade in breast cancer, gallbladder carcinoma, and colorectal cancer [24, 25]. To evaluate whether Nectin-4 could activate PI3K/AKT pathway in OS, we used GSVA analysis and GSEA analysis. As demonstrated in Additional file 4: Fig. S4B, GSVA analysis showed that the PI3K pathway was markedly enriched in the group of highly-expressed Nectin-4. In addition, using GSEA analysis, we also found that the PI3K pathway displays a positive correlation with Nectin-4 expression in the OS TARGET database (Additional file 4: Fig. S4C). To investigate whether Nectin-4 was involved in regulating OS-related EMT through the PI3K/AKT pathway, we observed the expression levels of AKT and p-AKT in both MG63 and U2OS cells. We found that the expression of p-AKT in the Nectin-4-OE group was much higher than that in the Vector-NC group. On the contrary, knockdown of Nectin-4 obviously decreased the expression of p-AKT in OS cell lines (Fig. 4C and Additional file 4: Fig. S4A). Besides, we also detected the up-regulation of p-NF-κB P65 (p-P65) in the Nectin-4-OE group compared with the Vector-NC group (Fig. 4C and Additional file 4: Fig. S4A). Based on these findings, we speculate that Nectin-4 might modulate the EMT pathway by regulating PI3K/AKT/ NF-κB pathway.

Subsequently, we managed to observe if we could adopt a classical PI3K inhibitor named LY294002 to reverse the effect of Nectin-4 overexpression. Our results revealed that LY294002 successfully reversed the Nectin-4 overexpression effect on the expression of markers of EMT and PI3K/AKT pathway (p-AKT) (Fig. 4D and Additional file 4: Fig. S4D). Moreover, to further validate whether Nectin-4 could regulate EMT markers by PI3K/AKT/NF-κB signal pathway in OS cell lines, we picked out a kind of NF-κB inhibitor (PDTC) to observe its effects on Nectin-4-OE MG63 and U2OS cells. The results demonstrated that PDTC could block Nectin-4 overexpression on EMT-related markers and p-P65 (Fig. 4E and Additional file 4: Fig. S4E). In addition, we detected that LY294002 could reverse the Nectin-4 overexpression effect on the expression levels of p-P65 (Fig. 4D). Functionally, we also revealed that LY294002 could reverse the function of Nectin-4 up-regulation on migratory and invasive activities in both MG63 and U2OS cell lines (Fig. 4F, both *P* < 0.01). Interestingly, we observed high AKT and P65 expression levels when Nectin-4 was up-regulated, while down-regulation of Nectin-4 led to the opposite effect. Above all, we speculated that Nectin-4 could modulate the levels of AKT and P65 to stimulate the PI3K/AKT/NF-κB signaling.

### MiR-520c-3p is a downstream regulator that targets AKT1 and P65

**Fig. 5:**
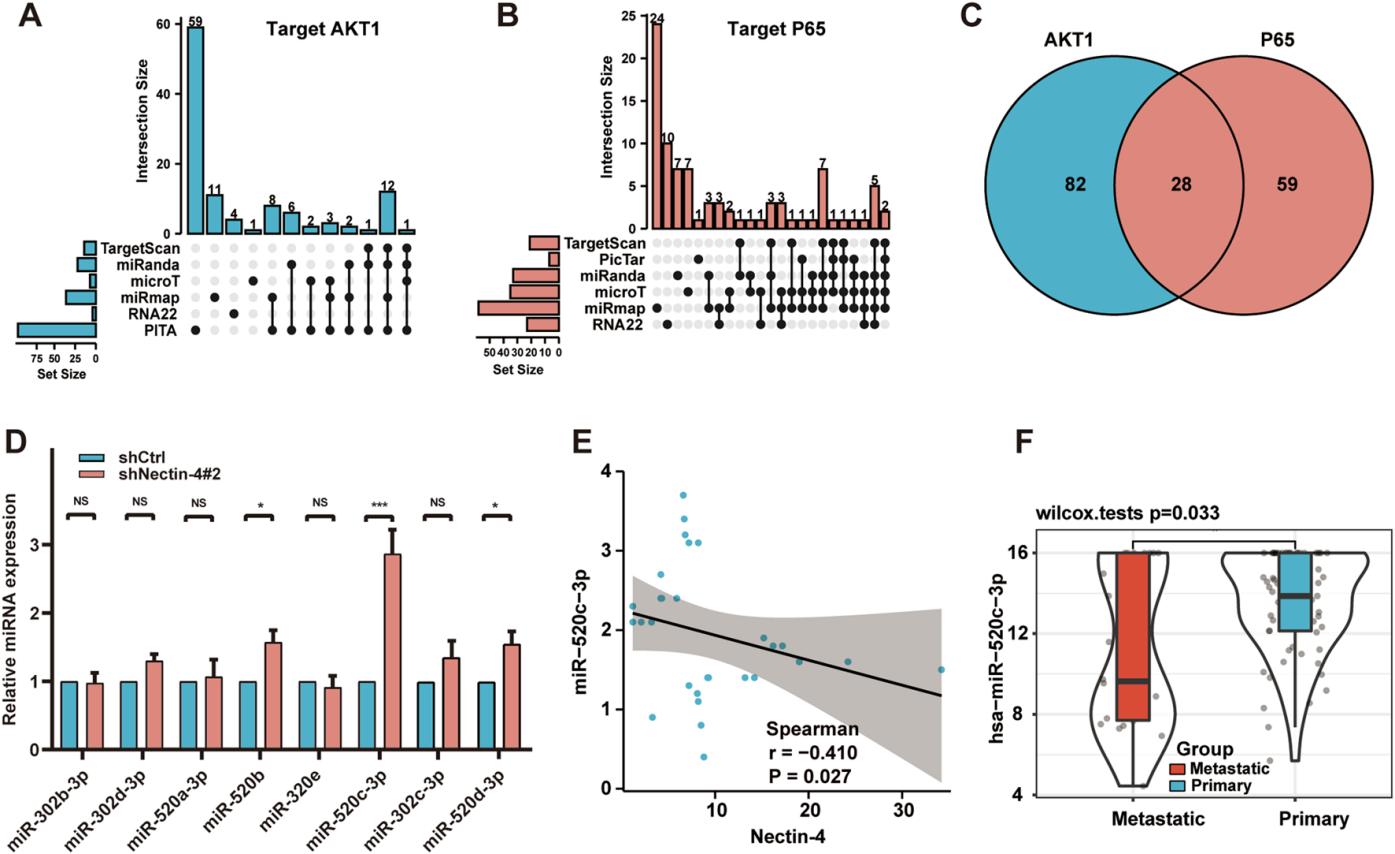
MiR-520c-3p is a downstream regulator that targets AKT1 and p65. **A**, **B** The potential miRNA targeting AKT1 or P65 were predicted by six online bioinformatics analysis software databases, including TargetScan, miRanda, microT, miRmap, RNA22, and PITA. **C** The miRNAs concurrent targeting AKT1 and p65 were generated by taking the intersection of the two miRNAs clusters using Venn diagrams. **D** The evaluation of these selected miRNAs expression levels was detected by using RT-qPCR in stable Nectin-4 knockdown 143B cells. **E** The Spearman′s correlation analysis was performed to discuss the relation between Nectin-4 and miR-520c-3p expression in OS tissues. **F** The differential expression of miR-520c-3p between primary and metastatic OS samples in the TARGET database. Each assay was repeated at least three times. ns, no significance; *P < 0.05; **P < 0.01; ***P < 0.001

Next, we wanted to clarify the mechanism more in depth through which Nectin-4 modulated PI3K/AKT/NF-κB signal pathway. Given that miRNAs could have significant regulatory roles by targeting mRNAs for cleavage or translational repression, we focused on the effects of Nectin-4-regulated miRNAs targeting AKT and NF-κB. As AKT1 has a crucial role in PI3K/AKT signaling in OS, P65 served as the most important subunit of the NF-κB complex [9–12]. Potential miRNA targeting AKT1 and P65 were predicted by six online bioinformatics analysis software databases, including TargetScan, miRanda, microT, miRmap, RNA22, and PITA (https://starbase.sysu.edu.cn/agoClipRNA.php?source=mRNA). As shown in Fig. 5A and B, 82 and 59 miRNAs targeting AKT1 and P65 were respectively selected after initial evaluation. Subsequently, we screened out 28 miRNAs simultaneously targeting AKT1 and P65, which were generated by taking the intersection of the two miRNAs clusters using Venn diagrams (Fig. 5C). Among them, we selected 12 miRNAs simultaneously predicted by at least three databases (Additional file 9: Table S1). Finally, we picked out 8 miRNAs (miR-302d-3p, miR-520d-3p, miR-302c-3p, miR-302b-3p, miR-520c-3p, miR-520a-3p, miR-520b, miR-302e) with relatively high reliability (target AKT1; context +  + score < -0.15 and weighted context +  + score < -0.15)in TargetScan software.

To assess which miRNA is the downstream regulator of Nectin-4, we evaluated these miRNAs expression levels by using RT-qPCR in stable Nectin-4 knockdown 143B cells, finding that miR-520c-3p was the microRNA with the most significantly up-regulated expression (≥ twofold) in Nectin-4 knockdown 143B cells (Fig. 5D). Moreover, the results of Spearman′s correlation analysis revealed that Nectin-4 expression had a significant negative correlation with the expression of miR-520c-3p in OS tissues (Fig. 5E), implying that miR-520c-3p might be negatively regulated by Nectin-4. Besides, the negative correlation between Nectin-4 and miR-520c-3p expression was also confirmed in the TARGET database (Additional file 5: Fig. S5A). In addition, we also detected the miR-502c-3p expression in 29 OS specimens and 8 adjacent normal specimens by using RT-qPCR. In both paired and unpaired specimens, the results revealed that the miR-520c-3p was significantly lower in OS specimens compared with adjacent normal specimens (Additional file 5: Fig. S5B and S5C). Furthermore, we attempted to analyze the differences in miR-520c-3p expression between primary and metastatic OS samples in TARGET database, finding that the expression of miR-520c-3p was significantly lower in OS metastatic samples than in primary samples (Fig. 5F). Therefore, we proposed that miR-520c-3p was regulated by Nectin-4, which was accordingly selected for follow-up studies.

### Nectin-4 activates PI3K/AKT/NF-κB signaling through modulating miR-520c-3p

**Fig. 6:**
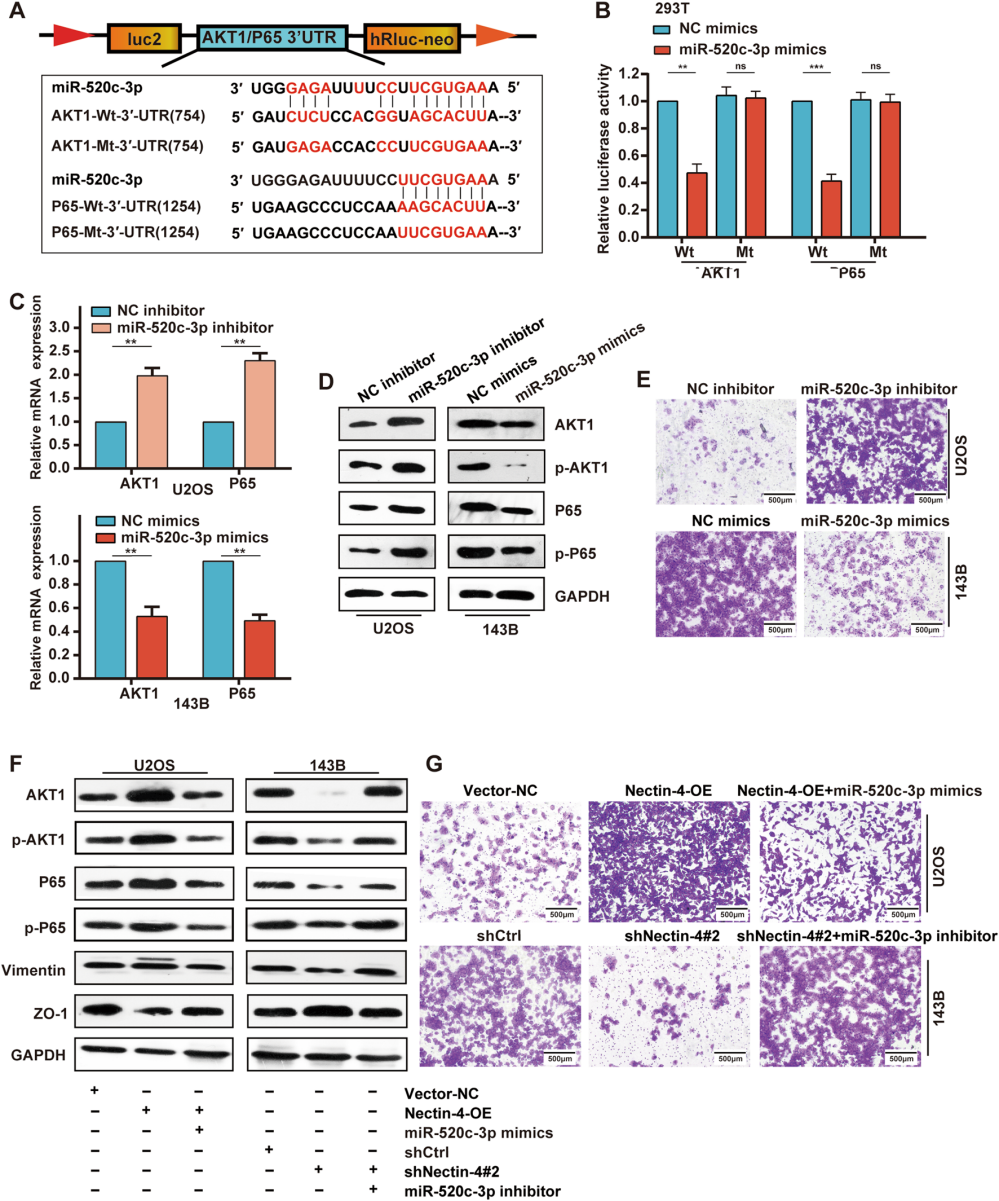
Nectin-4 activates PI3K/AKT/NF-κB signaling through modulating miR-520c-3p. **A** Schematic illustration of the complementary sequence between miR-520c-3p and AKT1(or P65). **B** Luciferase reporter vectors containing Wt or Mt AKT1 and P65 3′-UTR were constructed and co-transfected with miR-520c-3p mimics, or NC mimics into 293 T cells. Luciferase reporter assays used to determine whether miR-520c-3p directly binds to the 3′-UTR of AKT1 or P65. **C**, **D** The effects of miR-520c-3p silencing or overexpression on the expression of AKT, p-AKT, P65, and p-P65 in U2OS and143B cell lines by RT-qPCR and Western blotting, respectively. **E** The effects of miR-520c-3p silencing or overexpression on the migration ability in U2OS and 143B cell lines by transwell trials, respectively (scale bars 500 μm, magnifications of 100×). **F** The protein expression levels of EMT-related (ZO-1 and Vimentin) and PI3K/AKT/NF-κB pathway-related markers (AKT, p-AKT, P65, and p-P65) in Nectin-4-OE U2OS cells transfected with miR-520c-3p mimics, and in shNectin-4#2 143B cells transfected with miR-520c-3p inhibitor. **G** The migration ability of Nectin-4-OE U2OS cells transfected with miR-520c-3p mimics, and shNectin-4#2 143B cells transfected with miR-520c-3p inhibitor (scale bars 500 μm, magnifications of 100×). Each assay was repeated at least three times. ns, no significance; *P < 0.05; **P < 0.01; ***P < 0.001

According to the prediction analysis by TargetScan, we confirmed the binding sites of miR-520c-3p in the 3′-UTR of AKT1 and P65 mRNA (Fig. 6A). The luciferase reporters were co-transfected with miR-520c-sp mimic or inhibitor into HEK-293 T cells. We found that miR-520c-3p up-regulation significantly suppressed the luciferase activity of AKT1 and P65 with Wt 3′-UTRs, while down-regulation of miR-520c-3p led to the opposite effect. However, the miR-520c-3p mimics or inhibitor had no effect on those with Mt 3′-UTRs (Fig. 6B and Additional file 6: Fig. S6A). To clarify miR-520c-3p function in OS, as demonstrated in the Fig. S6B, we first assessed the basal expression of miR-520c-3p in different OS cell lines and osteoblastic cell lines by RT-qPCR. Subsequently, the 143B cells (lowest levels of miR-520c-3p) were transfected with miR-520c-3p mimics. Also, the MG63 and U2OS cells (higher levels of miR-520c-3p) were transfected with miR-520c-3p inhibitor. We measured the transduction efficiencies in the OS cell lines by using RT-qPCR (Additional file 6: Fig. S6C). Moreover, miR-520c-3p overexpression could significantly reduce the expression of AKT1, P65, p-AKT1, and p-P65 in 143B cells, as shown by RT-qPCR and Western blotting, respectively. miR-520c-3p knockdown resulted in the opposite results in the U2OS and MG63 cells (Fig. 6C, D and Additional file 6: Fig. S6D, S6E). In summary, these results demonstrated that AKT1 and P65 are direct targets of miR-520c-3p in OS cells.

As mentioned above, down-regulation of miR-520c-3p is closely related to OS metastases. To investigate the effect of miR-520c-3p on OS cell metastasis, we first detected the functional role of miR-520c-3p on the migratory capacity of OS cells by transwell assays, finding that much fewer migrated cells were observed in the miR-520c-3p mimics group cells compared to the NC mimics groups. Conversely, the migratory capacity in miR-520c-3p inhibitor group cells was remarkably improved compared with the NC inhibitor group (Fig. 6E and Additional file 6: Fig. S6F, S6G).

Next, to further clarify whether Nectin-4 could activate PI3K/AKT/NF-κB signaling mediated by miR-520c-3p in OS cells, miR-520c-3p mimics or miR-520c-3p inhibitor were transfected into Nectin-4-OE U2OS cells and shNectin-4 143B cells, respectively. The results of Western blotting trials demonstrated that the up-regulation of miR-520c-3p successfully reversed the Nectin-4 overexpression effect on the expression of markers of EMT (Vimentin and ZO-1) and PI3K/AKT/NF-κB pathway (AKT1, p-AKT1, P65, and p-P65) (Fig. 6F). On the contrary, the miR-520c-3p silencing reversed the Nectin-4 knockdown effect on the expression of markers of EMT and PI3K/AKT/NF-κB pathway (Fig. 6F). Consistently, the transwell trials also showed that miR-520c-3p overexpression reversed the function of Nectin-4 up-regulation on migratory activities in U2OS cells. Also, the knockdown of miR-520c-3p could reverse the effect of Nectin-4 silencing on migratory ability in 143B cells (Fig. 6G and Additional file 6: Fig. S6H). These findings confirmed that Nectin-4 could promote OS cells EMT and migration by activating PI3K/AKT/NF-κB signaling mediated by miR-520c-3p.

### Nectin-4 enhances OS cells tumorigenesis and lung metastasis in vivo

**Fig. 7:**
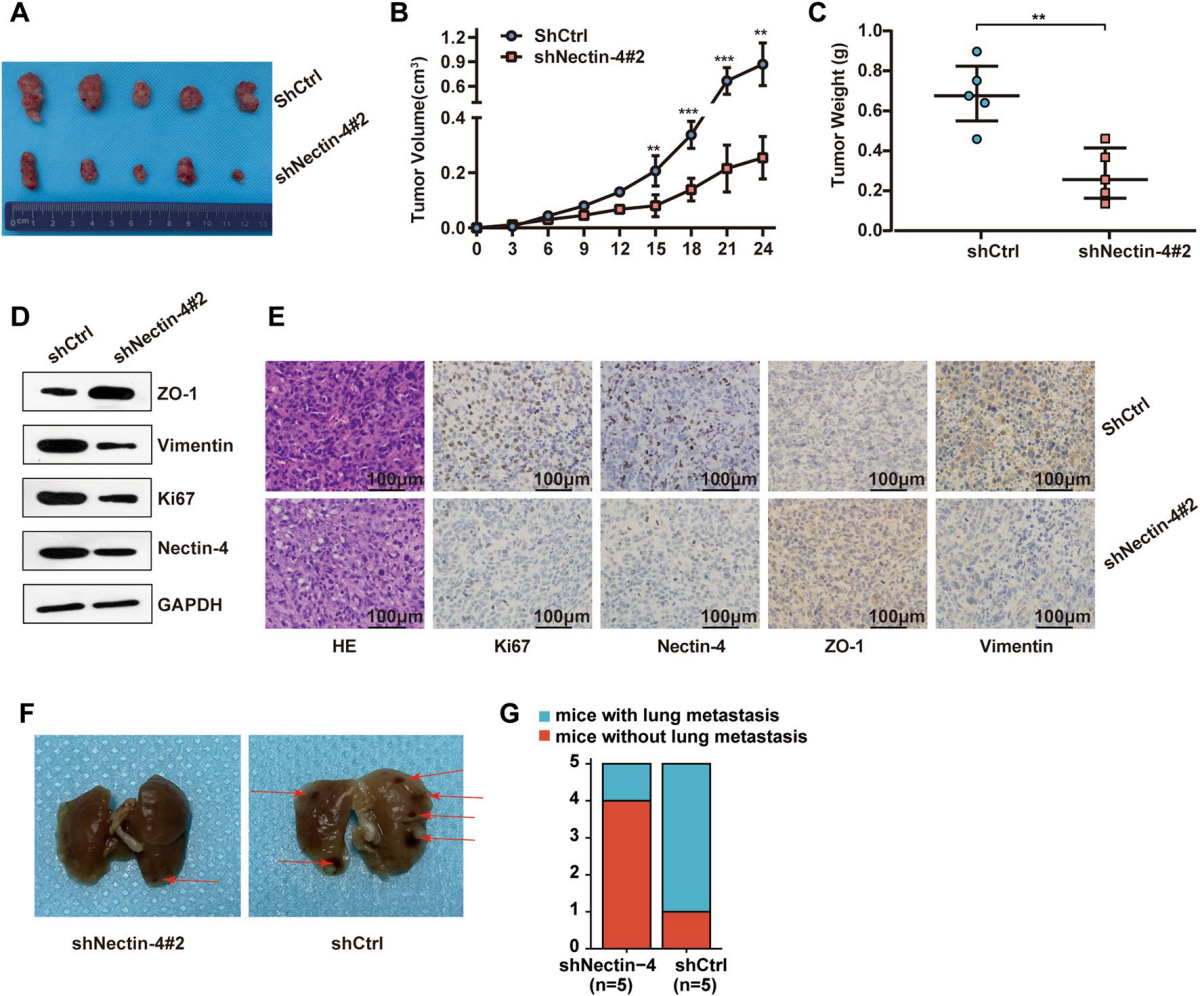
The effect of Nectin-4 knockdown on OS cells tumorigenesis and lung metastatic in vivo. **A** The Subcutaneous transplantation was successfully constructed after being injected with shNectin-4#2 or shCtrl 143B cells. The mice were sacrificed for tumor harvesting after 24 days and tumor image. **B** The tumor growth curves were plotted in the shNectin-4#2 group and shCtrl group. **C** The weight of tumors xenografts in the shNectin-4#2 group and the shCtrl group. **D** The protein expression of ZO-1, Vimentin, Ki67, Nectin-4 in the shNectin-4#2 group and the shCtrl group by using a Western blotting assay. **E** Hematoxylin–eosin (HE) staining and IHC staining for Ki67, Nectin-4, ZO-1, and Vimentin in shNectin-4#2 group and shCtrl group (scale bars 100 μm, magnifications of 200 ×). (**F**) Representative images of the lung metastasis model in the shNectin-4#2 group and shCtrl group. **G** The incidence of mice with lung metastasis in the shNectin-4#2 group and shCtrl group was statistically analyzed. The red arrow represented the lung tissue surface metastases. ns, no significance; *P < 0.05; **P < 0.01; ***P < 0.001

To directly evaluate the role of Nectin-4 in OS cells tumorigenesis and growth in vivo, we adopted the subcutaneous transplantation model of human OS cells in BALB/c-nude mice. Briefly, 143B cells transduced with shNectin-4#2 or shCtrl lenti-virus were subcutaneously injected into each flank of BALB/c-nude mice. Finally, all of the mice were killed to harvest the xenograft. The results indicated that the capacity of tumorigenesis in shNectin-4#2 mice group was remarkably lower than that in the shCtrl group (*P* < 0.01, Fig. 7A). Tumor growth of the shNectin-4#2 group was slower than that in the shCtrl group (Fig. 7B). Moreover, it was obvious that the mean weight of the subcutaneous tumors generated from the Nectin-4 down-regulation group was significantly lower compared with the control group (*P* < 0.01, Fig. 7C).

The total protein was extracted from the tumors, and the expression of Vimentin, Ki67, and Nectin-4 was more remarkably reduced in the shNectin-4#2 group than in the shCtrl group, while the expression of ZO-1 had the opposite trend (Fig. 7D). Additionally, HE staining for the tumor sections was performed to evaluate the tissue morphology, revealing much more cell nodules and masses in the shCtrl group than in shNectin-4#2 group (Fig. 7E). Meanwhile, the IHC staining also provided the same powerful evidence that the expression of Ki-67, Nectin-4, and Vimentin in Nectin-4 knockdown group was significantly decreased compared with the control group. Also, an opposite trend was observed in the expression of ZO-1 (Fig. 7E). Given these findings, we identified that the knockdown of Nectin-4 could markedly attenuate the OS cells tumorigenesis and growth in vivo.

Next, to further verify the crucial effect of Nectin-4 on the process of OS metastasis, we used a lung metastatic mouse model. Briefly, shNectin-4#2 or shCtrl 143B cells were injected into the five BALB/c-nude mice by lateral tail vein, respectively. After 3 weeks, the mice were anesthetized, and their lungs were dissected. As shown in Fig. 7F and G, based on visual observation, the quantities and incidence of mice with lung metastasis in shNectin-4#2 group mice were lower than those in the shCtrl group. These data clearly indicated that knockdown of Nectin-4 could prominently restrain the tumorigenesis and metastatic of OS in vivo.

To investigate whether Nectin-4 promoted tumorigenesis mediated by miR-520c-3p in vivo, we detected the functional role of miR-520c-3p knockdown in tumorigenesis based on Nectin-4 silencing. First, the shNectin-4#2 143B cells were transfected with miR-520c-3p inhibitor or NC inhibitor, after which they were subcutaneously injected into the flank of six nude mice (Additional file 7: Fig. S7A). Finally, the tumors were harvested from mice 24 days after injection. The tumors derived from the group of "shNectin-4#2 + miR-520c-3p inhibitor" had a much larger weight and volume than those from the group of "shNectin-4#2 + NC inhibitor" (Additional file 7: Fig. S7B and S7C). The results demonstrated that miR-520c-3p silencing significantly reversed the effect in tumorigenesis and growth caused by Nectin-4 silencing. Given these findings, we confirmed that Nectin-4 and miR-520c-3p had a vital role in promoting the proliferation and metastasis of OS in vivo.

## Discussion

Nectin-4 is a member of the Nectin family that contains four Ca^2+^-independent immunoglobulin-like cell adhesion molecules. It also has a key role in modulating cellular viability and movement capacity [13–16]. Importantly, an increasing number of studies have demonstrated that Nectin-4 is specifically overexpressed in various cancers [20–22]. For example, Challita-Eid et al. [35] collected more than two thousand tumor samples containing head/neck, lung, bladder, breast, pancreatic, ovarian, and esophageal tumors; in two-thirds of all specimens obviously high expression of Nectin-4 was detected by IHC staining. Moreover, a large number of studies demonstrated that overexpression of Nectin-4 served as a tumor-associated inducer in various malignant tumors, including colorectal, lung, pancreatic, ovarian, and breast cancers [18–23]. Until now, little is known about whether or not Nectin-4 exerts a vital influence on OS oncogenesis and development. Thereby, the aim of this study was to investigate the functional role and molecular mechanisms of Nectin-4 in human OS cells.

In our study, we firstly found that the mRNA and protein expression level of Nectin-4 displayed significant overexpression in OS tissues compared with adjacent normal tissues. In addition, we also found that the Nectin-4 high expression was markedly associated with tumor metastases in GSE21257. Moreover, the IHC results of OS TMA demonstrated that high expression of Nectin-4 was closely correlated with a higher OS stage. Unfortunately, the critical connection between Nectin-4 and clinical factors was not found in the TARGET-OS (Additional file 10: Table S2), which might be due to the limited OS tumor specimens. Hence, additional studies with a large number of samples are essential to further verify the connection between Nectin-4 and clinical factors in OS. Even so, numerous studies reported that up-regulation of Nectin-4 was correlated with the tumor progression and worse prognosis in various cancers [36–38]. Athanassiadou et al. [36] reported that overexpression of Nectin‑4 was correlated with tumor size, grade, and lymph nodes infiltration in breast cancer. In the study by Ma et al. [37], up-regulation of Nectin-4 was found to be correlated with TNM stage, tumor size, tumor spread and metastasis, and vascular involvement in hepatocellular carcinoma. In addition, Rabet and colleagues [38] demonstrated that patients who had higher expression of Nectin-4 were more likely to suffer a shorter life compared with those with down-regulation of Nectin-4 in triple-negative breast cancer (TNBC). Similarly, Takano [19] reported that nearly two-thirds of patients presented with up-regulation of Nectin-4 and had a very poor survival in lung cancer.

Based on the in vitro assays results, we concluded that up-regulation of Nectin-4 promoted human OS cells proliferation, migration, and invasion. In contrast with Nectin-4 high expression, the low expression of Nectin-4 exactly reversed the above effect in vitro. Similar results were also found in the study by Zhang et al. [39], which demonstrated that colorectal cancer (CRC) cells with Nectin-4 overexpression could facilitate the cells proliferation and migration, thus further enhancing the resistance to chemoradiotherapy. In addition, Zhang et al. (24) demonstrated that the low expression of Nectin‑4 restrained gall bladder cancer cell proliferation and migration both in vivo and in vitro*.* Furthermore, Nishiwada et al. (23) reported that knockdown of Nectin‑4 inhibited the proliferation of human pancreatic cancer cells. Similarly, Hao et al. (49) reported that Nectin-4 significantly promoted papillary thyroid cancer cell proliferation, migration, and invasion. The potential mechanism underlying Nectin-4 in promoting the tumor cells growth, proliferation, and movement was confirmed by regulating Ras-related C3 botulinum toxin substrate 1 (Rac1) signaling activity [40]. Rac1, as one of a member of the Rho family GTPases, exerted great influence on tumor occurrence and development [41]. Rac1 GTPase switched Rac1-GDP (“OFF” state) to a Rac1-GTP (“ON” state) [42, 43]. In addition, several researchers reported that elevated levels of Rac1 could be activated by the upstream modulator of PI3K/AKT in gallbladder carcinoma, gastric cancer, and breast cancer [44, 45].

EMT has been reported as the most critical cellular event before the occurrence of tumor migration, invasion, and metastasis [46]. Recent studies reported that Nectin-4 firstly combined with afadin and then regulated the actin cytoskeleton remodeling, which could induce EMT and enhance pseudopod driving force in tumor cell lines [47, 48]. In the recent research by Hao et al.[49], down-regulation of Nectin-4 in papillary thyroid cancer (PTC) cells suppressed EMT and markedly inhibited PTC cell migration and invasion via PI3K/AKT signal pathway. In the present study, we demonstrated that up-regulation of Nectin-4 contributes to a reduction of ZO-1 expression and notably elevates the expression of Vimentin and N-Cadherin. In contrast with Nectin-4 up-regulation, the down-regulation of Nectin-4 had the opposite effects on EMT-related markers. To investigate whether Nectin-4 could activate PI3K/AKT pathway in OS, we performed both GSVA analysis and GSEA analysis, finding that the PI3K pathway was markedly enriched in high expression of the Nectin-4 group. Moreover, we revealed that Nectin-4-OE group cells had higher p-AKT and p-P65 expression than those in the Vector-NC group. Subsequently, we found that PI3Kinhibitor (LY294002) and NF-κB inhibitor (PDTC) could reverse the impact of up-regulated Nectin-4 on the regulation of EMT-related markers and migration and invasion capacities.

**Fig. 8:**
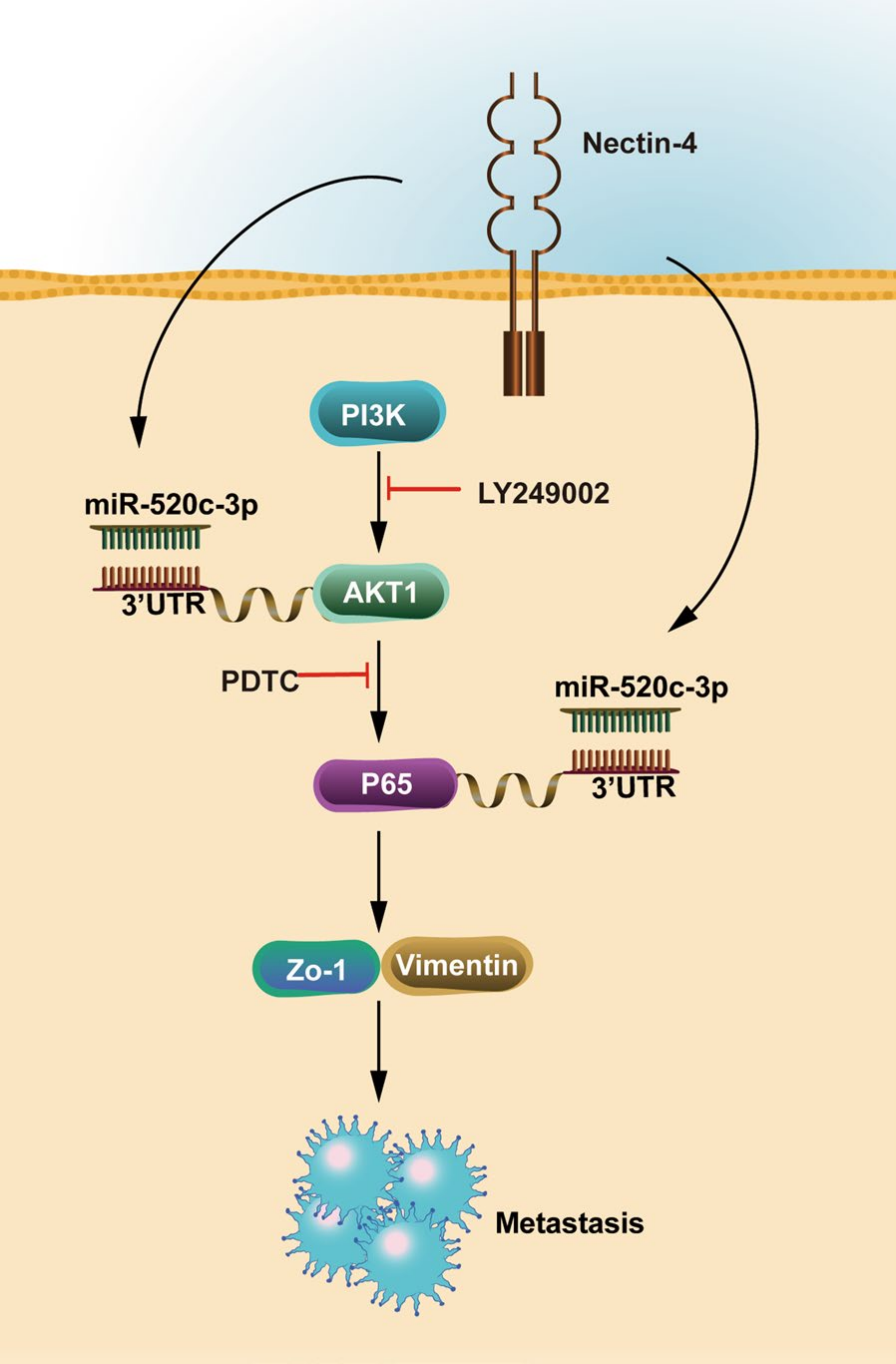
A proposed schematic model: the Nectin-4 regulates EMT via PI3K/AKT/NF-κB signal pathway mediated by miR-520c-3p

As the growing body of research suggests that microRNAs have a pivotal role in the progression and metastasis of OS by regulating target mRNAs [30, 50, 51], we aimed to find a novel miRNA to explain the underlying molecular mechanisms of OS development and progression development, after which we focused on the effects of Nectin-4-regulated miRNAs that targeted AKT and NF-κB. In our study, 8 potential miRNAs with relatively high reliability simultaneously targeting AKT1 and p65 were firstly selected by using six online bioinformatics analysis software databases. More importantly, miR-520c-3p was the microRNA with the most significantly up-regulated expression (≥ twofold) in stable Nectin-4 knockdown 143B cells by RT-qPCR. Subsequently, we observed that miR-520c-3p mimics and miR-520c-3p inhibitor could reverse the impact of Nectin-4 overexpression and silencing on the regulation of EMT-related markers and migration capacities, respectively. In addition, the luciferase reporter assay confirmed that miR-520c-3p could directly target AKT1 and P65. In view of these findings, we validated whether Nectin-4 could promote OS cells EMT and migration by activating PI3K/AKT/NF-κB signaling mediated by miR-520c-3p (Fig. 8). In our recent study, miR-520c-3p was differentially expressed in numerous cancers, including breast cancer, lung cancer, and colorectal cancer, and its expression was closely associated with cancer progression and prognosis in patients [32–34, 52], which is consistent with the results of the present study. Furthermore, the expression of miR-520c-3p was significantly lower in OS tissues than in normal tissues, and its low expression was positively associated with tumor metastasis.

Finally, we successfully built a subcutaneous transplantation model and lung metastatic mouse model. Our results indicated that knockdown of Nectin-4 could prominently reduce the tumorigenesis and metastasis of OS cells in vivo. Besides, we also found that miR-520c-3p silencing significantly reversed the effect in tumorigenesis and growth caused by Nectin-4 silencing. The above results confirmed that Nectin-4 promoted OS progression and metastasis by activating PI3K/AKT/NF-κB signaling through down-regulation of miR-520c-3p.

Recently, Enfortumab vedotin (EV) is a new type of antibody‑drug conjugates (ADCs) targeting Nectin‑4 in clinical practice [53, 54]. ADCs are novel monoclonal antibodies coupled with robust biological drugs via a labile crosslinker. Importantly, the antibody links with a specific antigen only found on target tumor cells. When the monoclonal antibody binds to antigen receptors of tumor cells, it triggers the internalization of the antibody and mediates drug release that could be viewed as ‘targeted chemotherapy’ [53, 54]. Clinically, EV was approved by the US Food and Drug Administration (FDA) in 2019 for treating locally advanced or metastatic UC (mUC) after the failure of previous chemotherapy regimens and immune checkpoint inhibitors (ICIs) [55]. As aforementioned, Nectin‑4 was a significantly marker specifically upregulated in OS, which promotes tumorigenesis and progression. Therefore, the EV targeting Nectin‑4 may be open a promising novel avenue for developing therapeutic strategies that could improve OS patient outcomes.

## Conclusion

Taken together, our present study firstly demonstrated the clear evidence that Nectin-4 is specifically up-regulated in OS tissues and cells, and its expression is closely related to tumor stage and metastasis. In addition, Nectin-4 promotes OS progression and metastasis by activating PI3K/AKT/NF-κB signaling mediated through miR-520c-3p. Therapeutically, Nectin-4 may serve as a target for the treatment of osteosarcoma.

## Supplementary Information

**Additional file 1: Figure S1.** The expression of Nectin-4 in OS tissues and normal tissues. (**A**) The differential expression of Nectin-4 in OS tissues and normal muscle tissues obtained from the TARGET and GTEx database, respectively. (**B**) The scatter plots of Nectin-4 IHC score for different OS stages and normal tissue. ns, no significance; *P<0.05; **P<0.01; ***P<0.001.

**Additional file 2: Figure S2.** The effect of Nectin-4 on human OS cells proliferation. (A) The results of Western blotting assay for the up-regulation of Nectin-4 in human MG63 and U2OS cells. (B) The results of Western blotting for the effectiveness of shNectin-4#1, #2, and #3 at the protein level in 143B cells. (C) The results of the Colony formation in MG63, U2OS cells (infected with Vector-NC or Nectin-4-OE lentivirus), and 143B cells (infected with shCtrl or shNectin-4#2 lentivirus). Each assay was repeated at least three times. ns, no significance; *P<0.05; **P<0.01; ***P<0.001

**Additional file 3: Figure S3.** The migration capacity of the MG63, U2OS cells (Nectin-4-OE group vs. Vector-NC group), and 143B (shNectin-4#2 group vs. shCtrl group) performed by wound healing assay. Each assay was repeated at least three times. ns, no significance; *P<0.05, **P<0.01, ***P<0.001.

**Additional file 4: Figure S4.** Nectin-4 modulates EMT and migration potency via PI3K/AKT/NF-κB signal pathway. (A) The results of quantitative analysis of Western blotting for the influence of Nectin-4 on the expression of EMT-related markers and PI3K/AKT/NF-κB pathway-related markers in 143B, MG63, and U2OS cell lines (infected with Nectin-4-OE, Vector-NC, shCtrl, and shNectin-4#2 Lentivirus, respectively) by. (B, C) By using GSVA and GSEA analysis, we detected the relationship between the high expression of Nectin-4 and the activation of the PI3K pathway, respectively. (D) The results of quantitative analysis for the protein expression levels of EMT-related and PI3K/AKT pathway-related markers in Nectin-4-OE U2OS and MG63 cell lines treated with DMSO and LY294002 (PI3K inhibitor). (E) The results of quantitative analysis for the protein expression levels of EMT-related and PI3K/AKT pathway-related markers in Nectin-4-OE U2OS and MG63 cell lines treated with DMSO and PDTC (NF-κB inhibitor). Each assay was repeated at least three times. ns, no significance; *P<0.05; **P<0.01; ***P<0.001.

**Additional** **file** **5:** **Figure** **S5**. The correlation between Nectin-4 and miR-520c-3p expression and the differential expression of miR-502c-3p between OS and normal specimens. (A) The Spearman′s correlation analysis was performed to discuss the relation between Nectin-4 and miR-520c-3p expression by the integration of RNA-seq and miRNAs data from the TARGET database. (B) The miR-502c-3p expression in 29 OS specimens and 8 adjacent normal specimens by using RT-qPCR. (C) The miR-502c-3p expression in 8 paired OS specimens and 8 adjacent normal specimens by using RT-qPCR. ns, no significance; *P<0.05; **P<0.01; ***P<0.001.

**Additional file 6: Figure S6.** Nectin-4 activates PI3K/AKT/NF-κB signaling mediated by miR-520c-3p. (A) Luciferase reporter vectors containing Wt or Mt AKT1 and P65 3′-UTR were constructed and co-transfected with miR-520c-3p inhibitor or NC inhibitor into 293T cells. (B) The basal expression levels of miR-520c-3p in OS cell lines (MG63, U2OS, and 143B) and osteoblastic cell line (hFOB1.19) by using RT-qPCR. (C) RT-qPCR analysis of the expression of miR-520c-3p in U2OS, MG63, and 143B cells respectively transfected with miR-520c-3p inhibitor, or NC inhibitor, or miR-520c-3p mimic, or NC mimics. (D, E) The effects of miR-520c-3p silencing on the expression of AKT, P65, p-AKT, and p-P65 in MG63 cells by RT-qPCR and Western blotting, respectively. (F) The effects of miR-520c-3p silencing on the migration ability in MG63 cells by transwell trials (scale bars 500μm, magnifications of 100×). (G) The effects of miR-520c-3p overexpression or silencing on the migration ability in 143B, MG63, and U2OS cell lines by transwell trials. (H) The migration ability of Nectin-4-OE U2OS cells transfected with miR-520c-3p mimics and shNectin-4#2 143B cells transfected with miR-520c-3p inhibitor. Each assay was repeated at least three times. ns, no significance; *P<0.05; **P<0.01; ***P<0.001.

**Additional file 7: Figure S7.** The effect of miR-520c-3p knockdown on shNectin-4#2 OS cells tumorigenesis in vivo. (A) The Subcutaneous transplantation was successfully constructed after being injected with shNectin-4#2 143B cells transfected with miR-520c-3p inhibitor or NC inhibitor. The mice were sacrificed for tumor harvesting after 24 days and tumor image. (B) The tumor growth curves of the groups of the shNectin-4#2 143B cells were transfected with miR-520c-3p inhibitor or NC inhibitor. (C) The weight of tumor xenografts in the groups of the shNectin-4#2 143B cells was transfected with miR-520c-3p inhibitor or NC inhibitor. ns, no significance; *P<0.05; **P<0.01; ***P<0.001

**Additional file 8**: Materials and Methods

**Additional file 9: Table S1.** Potential mircoRNAs targeting both AKT1 and P65 were predicted by six online bioinformatics analysis software databases by using Venn diagrams.

**Additional file 10: Table S2**. Association between Nectin-4 expression and clinicopathologic features in TARGET

**Additional file 11: Table S3.** Primer sequences for quantitative real-time PCR and shRNA directing at the human Nectin-4 sequence

## Data Availability

All data presented are provided freely in this manuscript including any Supplementary data.
