# Supplementary figures and images for: Nectin-4 promotes osteosarcoma progression and metastasis through activating PI3K/AKT/NF-κB signaling by down-regulation of miR-520c-3p
Source: Cancer Cell Int. 2022 Aug 11;22:252. doi: 10.1186/s12935-022-02669-w (PMC9367085; doi:10.1186/s12935-022-02669-w)

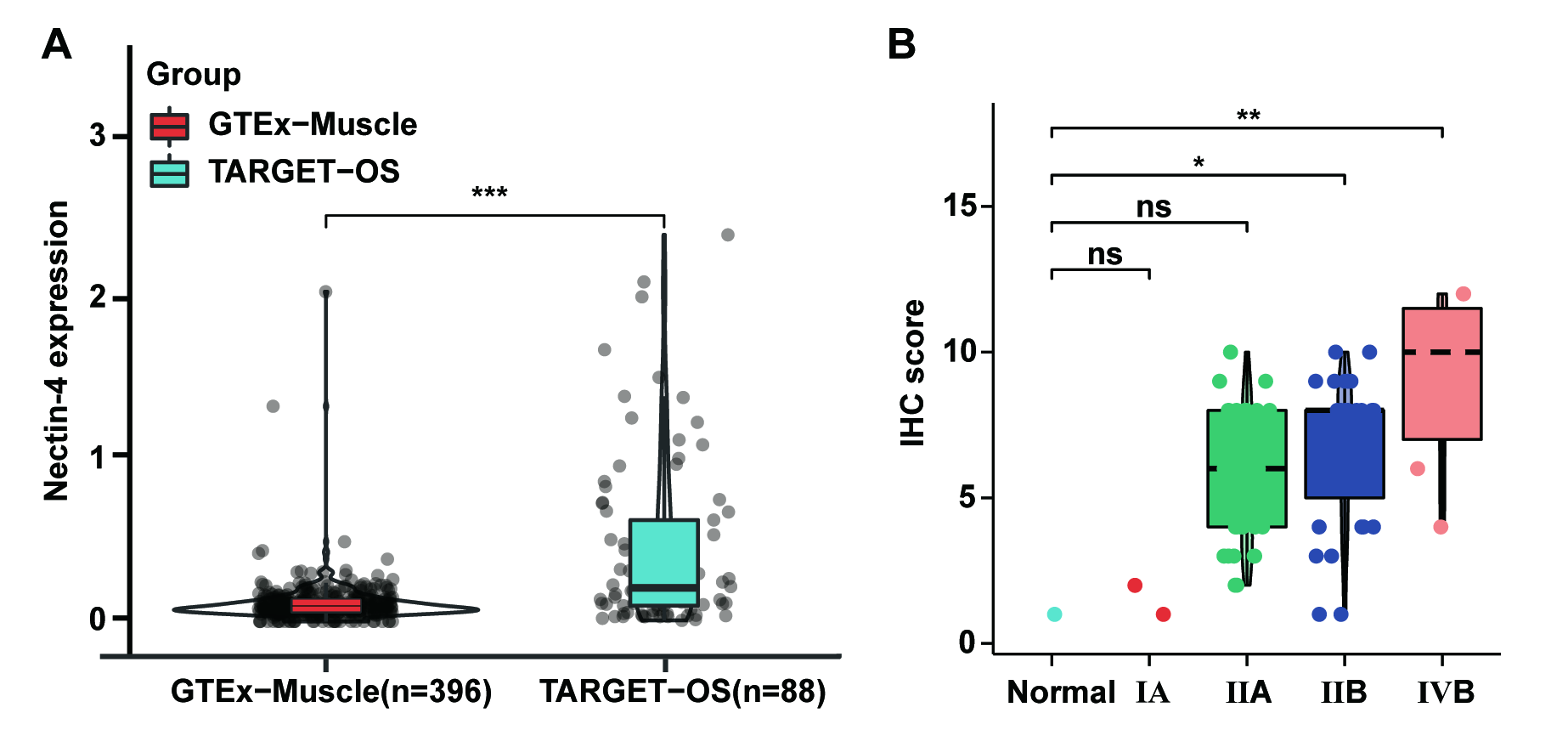

Supplement: Supplementary file 1 — Additional file 1: Figure S1. The expression of Nectin-4 in OS tissues and normal tissues. (A) The differential expression of Nectin-4 in OS tissues and normal muscle tissues obtained from the TARGET and GTEx database, respectively. (B) The scatter plots of Nectin-4 IHC score for different OS stages and normal tissue. ns, no significance; *P<0.05; **P<0.01; ***P<0.001. [file 12935_2022_2669_MOESM1_ESM.tif]

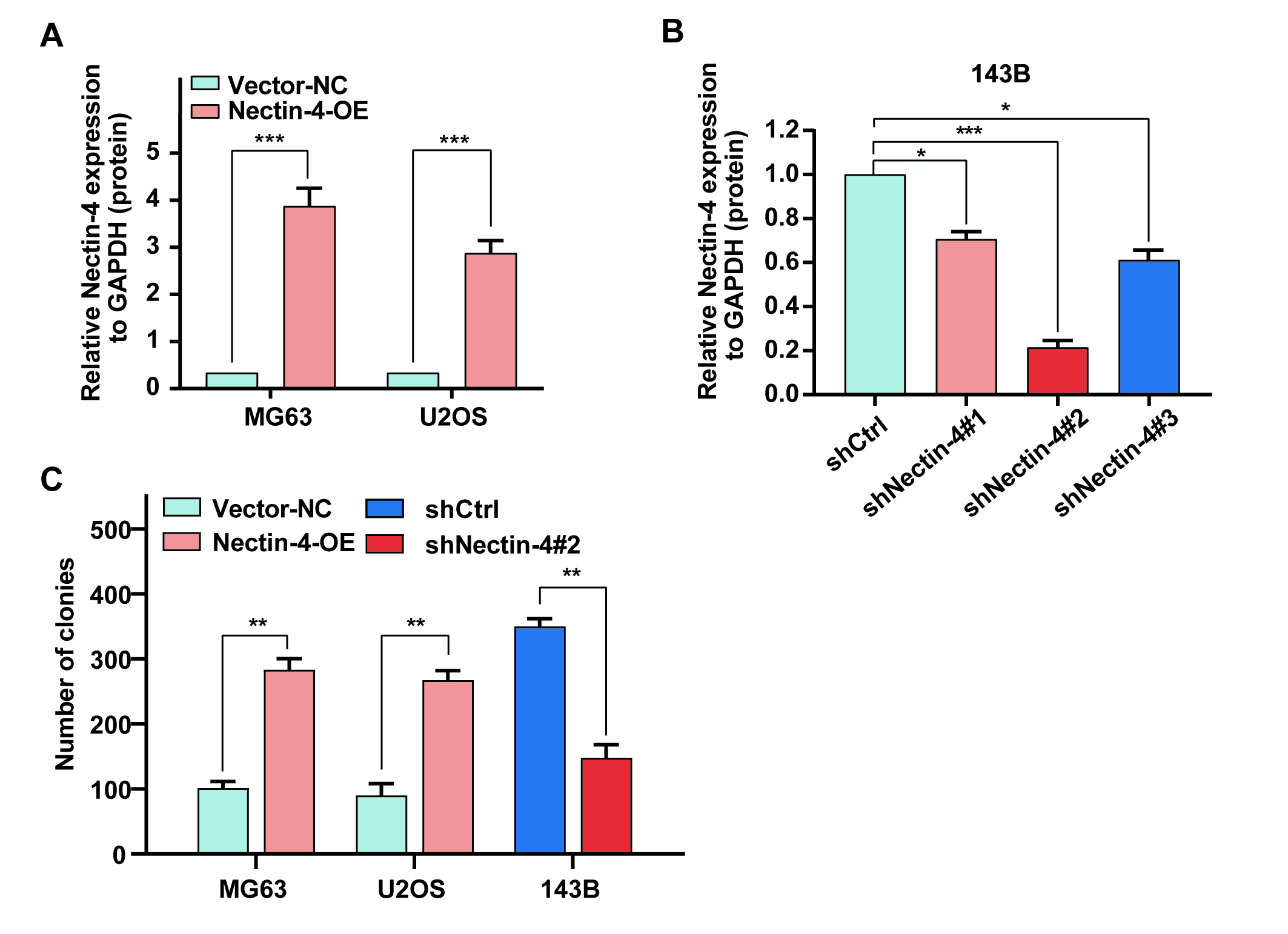

Supplement: Supplementary file 2 — Additional file 2: Figure S2. The effect of Nectin-4 on human OS cells proliferation. (A) The results of Western blotting assay for the up-regulation of Nectin-4 in human MG63 and U2OS cells. (B) The results of Western blotting for the effectiveness of shNectin-4#1, #2, and #3 at the protein level in 143B cells. (C) The results of the Colony formation in MG63, U2OS cells (infected with Vector-NC or Nectin-4-OE lentivirus), and 143B cells (infected with shCtrl or shNectin-4#2 lentivirus). Each assay was repeated at least three times. ns, no significance; *P<0.05; **P<0.01; ***P<0.001 [file 12935_2022_2669_MOESM2_ESM.tif]

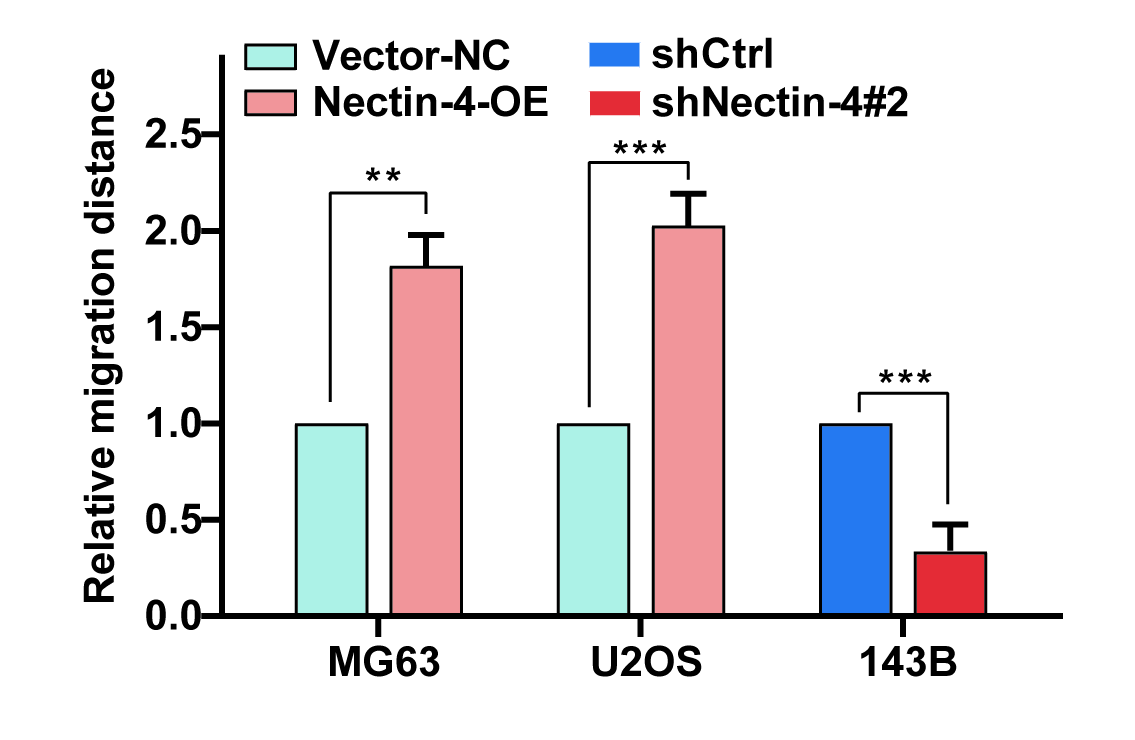

Supplement: Supplementary file 3 — Additional file 3: Figure S3. The migration capacity of the MG63, U2OS cells (Nectin-4-OE group vs. Vector-NC group), and 143B (shNectin-4#2 group vs. shCtrl group) performed by wound healing assay. Each assay was repeated at least three times. ns, no significance; *P<0.05, **P<0.01, ***P<0.001. [file 12935_2022_2669_MOESM3_ESM.tif]

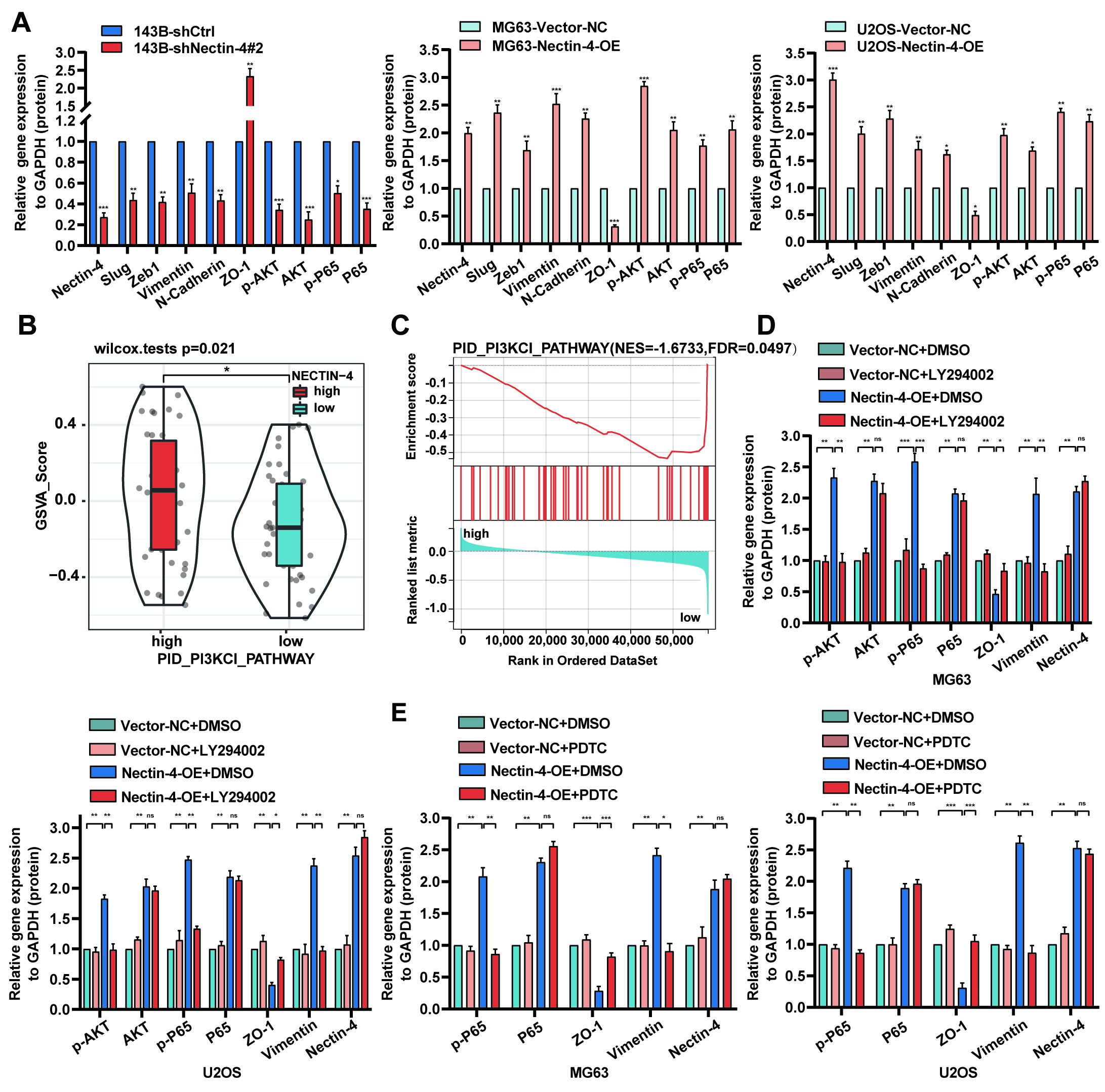

Supplement: Supplementary file 4 — Additional file 4: Figure S4. Nectin-4 modulates EMT and migration potency via PI3K/AKT/NF-κB signal pathway. (A) The results of quantitative analysis of Western blotting for the influence of Nectin-4 on the expression of EMT-related markers and PI3K/AKT/NF-κB pathway-related markers in 143B, MG63, and U2OS cell lines (infected with Nectin-4-OE, Vector-NC, shCtrl, and shNectin-4#2 Lentivirus, respectively) by. (B, C) By using GSVA and GSEA analysis, we detected the relationship between the high expression of Nectin-4 and the activation of the PI3K pathway, respectively. (D) The results of quantitative analysis for the protein expression levels of EMT-related and PI3K/AKT pathway-related markers in Nectin-4-OE U2OS and MG63 cell lines treated with DMSO and LY294002 (PI3K inhibitor). (E) The results of quantitative analysis for the protein expression levels of EMT-related and PI3K/AKT pathway-related markers in Nectin-4-OE U2OS and MG63 cell lines treated with DMSO and PDTC (NF-κB inhibitor). Each assay was repeated at least three times. ns, no significance; *P<0.05; **P<0.01; ***P<0.001. [file 12935_2022_2669_MOESM4_ESM.tif]

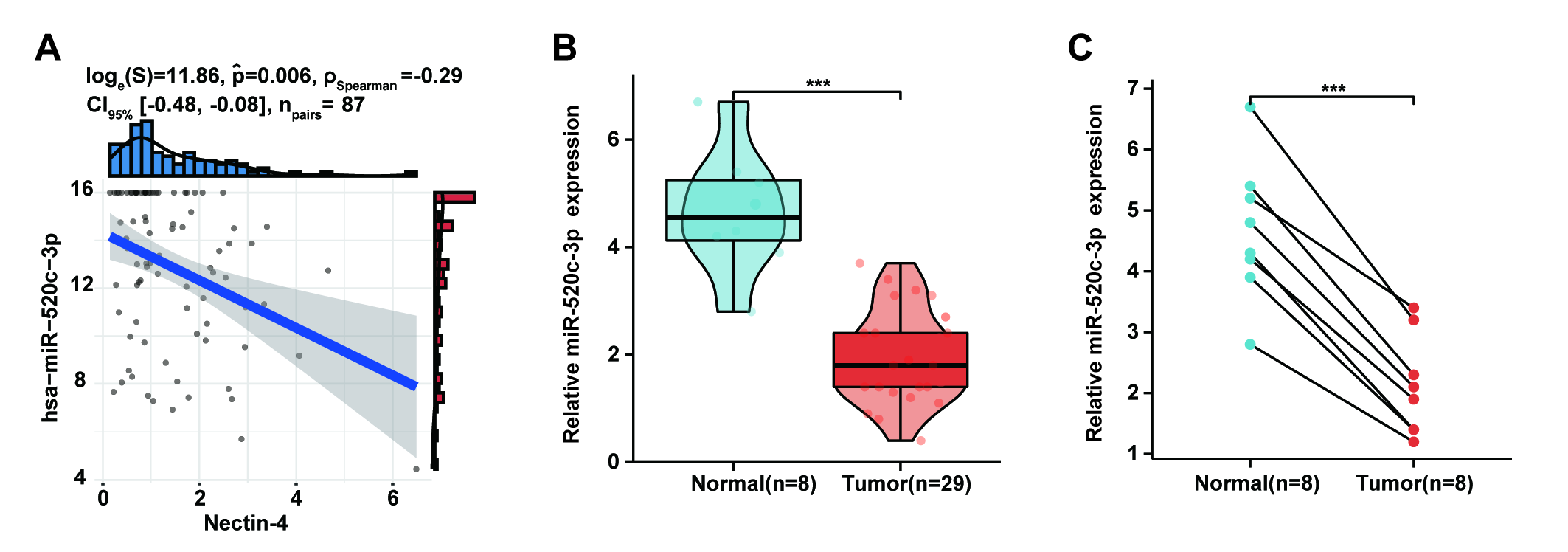

Supplement: Supplementary file 5 — Additional file 5: Figure S5. The correlation between Nectin-4 and miR-520c-3p expression and the differential expression of miR-502c-3p between OS and normal specimens. (A) The Spearman′s correlation analysis was performed to discuss the relation between Nectin-4 and miR-520c-3p expression by the integration of RNA-seq and miRNAs data from the TARGET database. (B) The miR-502c-3p expression in 29 OS specimens and 8 adjacent normal specimens by using RT-qPCR. (C) The miR-502c-3p expression in 8 paired OS specimens and 8 adjacent normal specimens by using RT-qPCR. ns, no significance; *P<0.05; **P<0.01; ***P<0.001. [file 12935_2022_2669_MOESM5_ESM.tif]

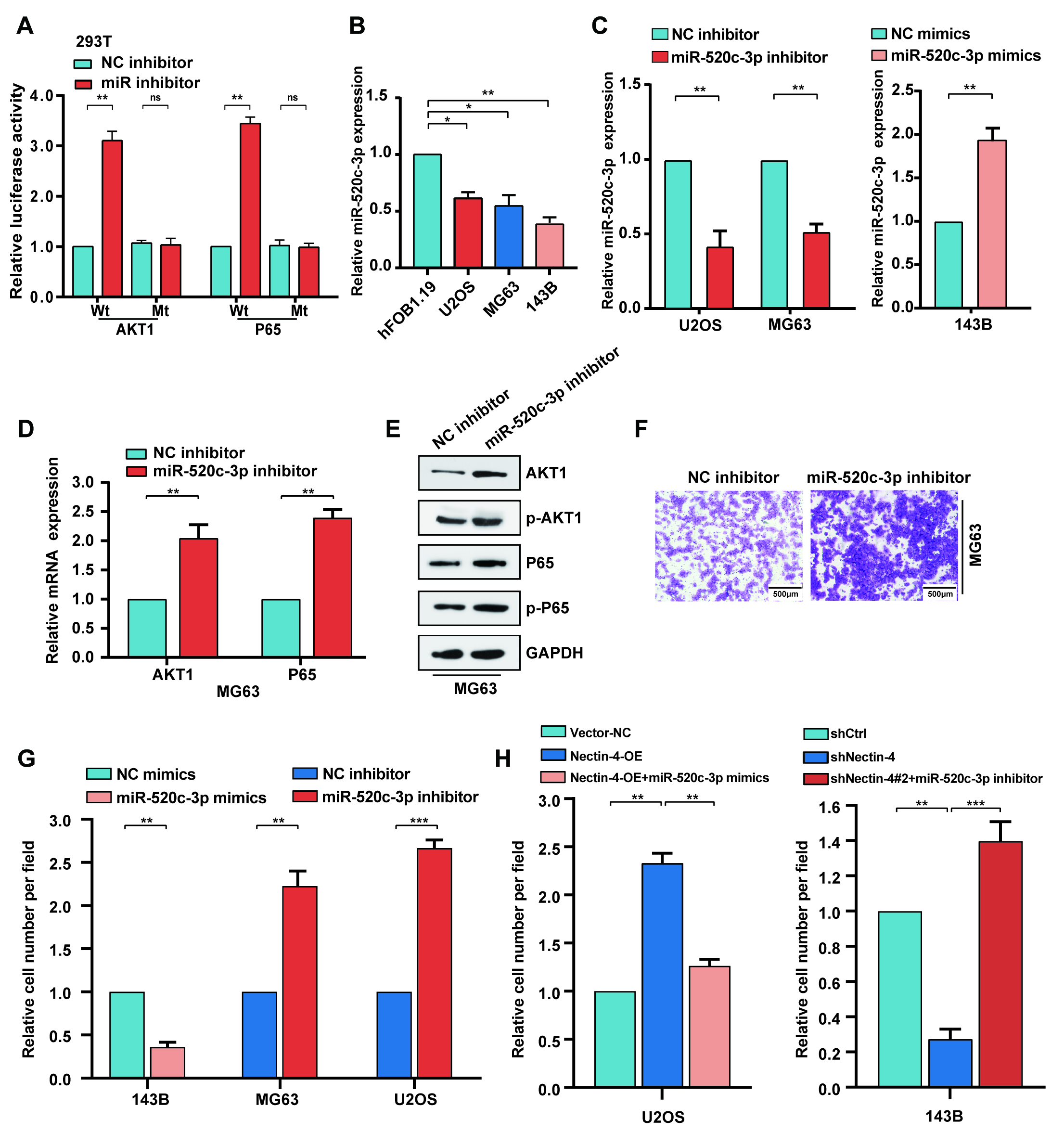

Supplement: Supplementary file 6 — Additional file 6: Figure S6. Nectin-4 activates PI3K/AKT/NF-κB signaling mediated by miR-520c-3p. (A) Luciferase reporter vectors containing Wt or Mt AKT1 and P65 3′-UTR were constructed and co-transfected with miR-520c-3p inhibitor or NC inhibitor into 293T cells. (B) The basal expression levels of miR-520c-3p in OS cell lines (MG63, U2OS, and 143B) and osteoblastic cell line (hFOB1.19) by using RT-qPCR. (C) RT-qPCR analysis of the expression of miR-520c-3p in U2OS, MG63, and 143B cells respectively transfected with miR-520c-3p inhibitor, or NC inhibitor, or miR-520c-3p mimic, or NC mimics. (D, E) The effects of miR-520c-3p silencing on the expression of AKT, P65, p-AKT, and p-P65 in MG63 cells by RT-qPCR and Western blotting, respectively. (F) The effects of miR-520c-3p silencing on the migration ability in MG63 cells by transwell trials (scale bars 500μm, magnifications of 100×). (G) The effects of miR-520c-3p overexpression or silencing on the migration ability in 143B, MG63, and U2OS cell lines by transwell trials. (H) The migration ability of Nectin-4-OE U2OS cells transfected with miR-520c-3p mimics and shNectin-4#2 143B cells transfected with miR-520c-3p inhibitor. Each assay was repeated at least three times. ns, no significance; *P<0.05; **P<0.01; ***P<0.001. [file 12935_2022_2669_MOESM6_ESM.tif]

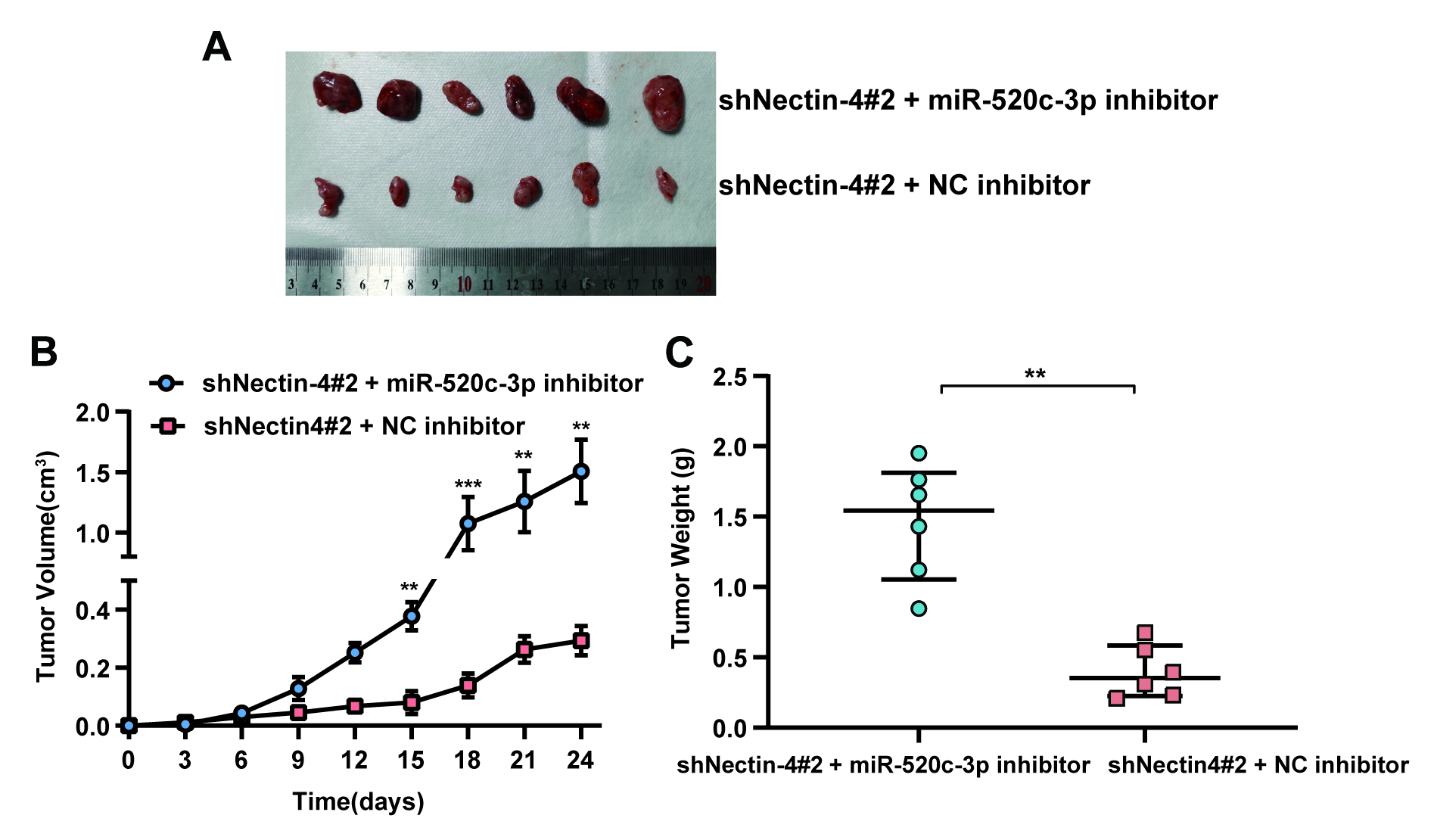

Supplement: Supplementary file 7 — Additional file 7: Figure S7. The effect of miR-520c-3p knockdown on shNectin-4#2 OS cells tumorigenesis in vivo. (A) The Subcutaneous transplantation was successfully constructed after being injected with shNectin-4#2 143B cells transfected with miR-520c-3p inhibitor or NC inhibitor. The mice were sacrificed for tumor harvesting after 24 days and tumor image. (B) The tumor growth curves of the groups of the shNectin-4#2 143B cells were transfected with miR-520c-3p inhibitor or NC inhibitor. (C) The weight of tumor xenografts in the groups of the shNectin-4#2 143B cells was transfected with miR-520c-3p inhibitor or NC inhibitor. ns, no significance; *P<0.05; **P<0.01; ***P<0.001 [file 12935_2022_2669_MOESM7_ESM.tif]
