# Supplementary material for: Nectin-4 promotes osteosarcoma progression and metastasis through activating PI3K/AKT/NF-κB signaling by down-regulation of miR-520c-3p
Source: Cancer Cell Int. 2022 Aug 11;22:252. doi: 10.1186/s12935-022-02669-w (PMC9367085; doi:10.1186/s12935-022-02669-w)
Supplement: Supplementary file 8 — Additional file 8: Materials and Methods [file 12935_2022_2669_MOESM8_ESM.docx]

**Supplementary Materials and Methods**

**Immunohistochemistry**

According to the manufacturer's protocols, TMAs and tumor sections (obtained from mice tumor tissues) were firstly dewaxed with xylene and dehydrated using gradient ethanol, after which they were subjected to EDTA antigen retrieval (PH9.0, 1mmol/l, 95˚C for 15 min). Endogenous peroxidase was blocked with Endogenous Peroxidase Blocking Buffer (cat.no. P0100A; Beyotime) for 10 min at room temperature, and then, the tissues were incubated with 3% Bovine Serum Albumin (cat.no.ST025; Beyotime) for 10 min. Subsequently, tissues were incubated overnight with primary antibody at 4˚Cand then with biotinylated goat anti‑mouse/rabbit IgG secondary antibody (cat.no. A0286; Beyotime) for 10 min at room temperature. Next, the tissues were incubated with streptavidin‑peroxidase for 10 min. Finally, the tissues were stained with diaminobenzidine (DAB)/ACE (cat.no. P0202; Beyotime) for 5 min and re-dyed with Hematoxylin Staining Solution (cat.no. C0107; Beyotime) for 3 min. The methods for simi-quantitative evaluation of the immunohistochemistry results are as following:

1. The percentage score: The lowest score was 0 and the highest score was 4 according to the percentage of antibody positive staining cells.

0 score: no positive staining cells were found.

1 score: the percentage of positive staining cells＜10%;

2 score: 10%≤the percentage of positive staining cells＜50%;

3 score: 50%≤the percentage of positive staining cells＜80%;

4 score: the percentage of cells with positive staining cells > 80%.

1. The intensity score: The score was given according to the intensity of positive staining cells, with the lowest score being 0 and the highest score being 3:

0 score: no positive staining cells;

1 score: weakly positive staining cells;

2 score: moderately positive staining cells;

3 score: strongly positive staining cells.

1. At last, the final sample staining score = (the percentage score) **╳** (the intensity score) (Select 5 visual fields for each slice and takes the average of staining scores). The final score was divided into low expression (final score≤6) and high expression (score＞6).

**Data Sources and Preprocessing**

We also collected the corresponding public datasets containing >50 OS samples from Gene Expression Omnibus (GEO) database and the Therapeutically Applicable Research To Generate Effective Treatments (TARGET) database. The TARGET-OS (High Throughput Sequence fragments per kilobase million, HTSeq- FPKM) includes 88 OS samples with miRNA, RNA sequencing (RNA-Seq) information, and clinical parameters, which were obtained from UCSC Xena (https://xenabrowser.net/datapages/). Moreover, ID/Gene conversion and annotation were performed through gencode.v22.annotation.gene.probeMap (https://gdc-hub.s3.us-east-.amazonaws.com/download/gencode.v22.annotation.gene.probeMap). Moreover, we also collected the gene expression data of Nectin-4 in normal muscular tissues, which were downloaded from the Genotype-Tissue Expression (GTEx) Muscular database (https://xenabrowser.net/datapages/). In addition, 53 OS samples with the gene expression profile of Nectin-4 and clinical parameters in the GSE21257 cohort (GPL10295 Illumina human-6 v2.0 expression beadchip) were downloaded from the GEO database (https://www.ncbi.nlm.nih.gov/geo/).

**Materials**

All chemicals were acquired commercially. High-glucose (HG) Dulbecco's Modified Eagle Medium (HG-DMEM) was obtained from Gibco-BRL (NY, USA). The primary antibodies for Western blotting or IHC were purchased from Cell Signaling Technology (CST; USA, Bostin) and Abcam Company (Cambridge, UK) including Nectin-4 (abcam, ab192033), Vimentin (CST, #5741), N-Cadherin (CST, #13116), Claudin (CST, #13255), β-Catenin (CST, #8480), ZO-1 (abcam, ab216880), Snail (CST, #3879), Slug (CST, #9585), ZEB1 (CST, #3396), GAPDH (CST, #5174), AKT (CST, #4685), Phospho (p)-AKT(CST, #4060), NF-κB P65 (CST, #8801), p-NF-κB P65 (CST, #3033), AKT1 (CST, #2938), p-AKT1 (CST, #9018), Ki67(abcam, ab16667) and secondary antibodies (anti‑rabbit IgG; CST, #7074). The pathway inhibitor (LY294002 and PDTC) was purchased from MedChem Express (MCE, USA). We used 10 mM for both LY294002 and PDTC and dissolved it in DMSO. MiR-502-3p mimic and miR-502c-3p inhibitor were purchased from Sangon Biotech Shanghai Co., Ltd. (Shanghai, China). Cells were transiently transfected with 50 nM mimics or 50 nM inhibitor using Lipofectamine 2000 (Invitrogen).

**Cell lines and cell culture**

The hFOB1.19, MG63, U2OS, 143B, and HEK 293T cell lines were purchased from American Type Culture Collection (Manassas, VA, USA). All cells were cultured and maintained in HG-DMEM. Both culture mediums contained 10% fetal bovine serum (FBS), 100 U/ml penicillin, and 100 µg/ml streptomycin (both from Gibco; Fisher Scientific, Inc., Waltham, MA, USA). Both of those cells were cultured in six-well/6cm/10cm cell culture dish plates at concentrations of 5×10^5^, 5×10^6^, and 10^7^ cells/well, respectively, after which they were cultured at 37˚C in a circumstance involving 5% CO_2_.

**Packaging and infection of lentivirus vector**

The full-length Nectin-4 (NM_030916.2; GenBank) fragment was synthesized by ^®^Sangon Biotech (Shanghai, China) and cloned into pCDH lentiviral vector (System Biosciences, Mountain View, CA, USA). To knockdown Nectin-4 expression, the small hairpin RNA (shRNA) directed at the human Nectin-4 sequence (shNectin-4#1, shNectin-4#2 and shNectin-4#3; Additional file 11: Table S3) and negative control vector sequence (shCtrl; Additional file 11: Table S3), which were acquired from GeneChem (Shanghai, China), were cloned into pLKO.1 vector (Sigma, USA).

The lentiviral vectors of pCDH-Vector/Nectin-4 and pLKO.1-shCtrl/shNectin-4 were co-infected with the packaging plasmids psPAX2 (Addgene, Inc.), and the envelope plasmid pMD2.G (Addgene, Inc.) into 293T cells (ATCC; Manassas, VA, USA) using Lipofectamine® 2000 (Invitrogen Life Technologies, Carlsbad, CA, USA). After 6 h of incubation at 37˚C in a humidified atmosphere containing 5% CO_2_, DMEM was replaced with a complete medium (containing 10% FBS, Gibco; Thermo Fisher Scientific, Inc.). The supernatant was harvested after culturing for 48h and concentrated by a 0.45μm PES filter. MG63 and U2OS cells were infected with packaged lentivirus supernatant of pCDH-Vector/Nectin-4. Also, 143B cells were infected with packaged lentivirus supernatant of pLKO.1-shCtrl/shNectin-4. The medium was replaced with fresh medium 24 h post-infection, and cells were collected 72 h post-infection for subsequent stable clones screening. Gene transfection efficiency was verified by real-time quantitative PCR (RT-qPCR) and Western blotting.

**Thermo RT-qPCR analysis**

Total RNAs were isolated and extracted from OS cell lines (or OS tissues) using TRIzol® reagent (Invitrogen, Carlsbad, CA) according to the producer’s instructions. MiRcute miRNA first-strand cDNA synthesis kit (Tiangen Biotech, Beijing, China) and Primer-Script TM one-step RT-PCR kit (Takara Bio, Inc.) were used to reverse transcribe the total RNA of miRNA and mRNA into cDNA, respectively. RT-qPCR analysis was carried out in the StepOnePlus system (Applied Biosystems) and adopted SYBR-Green qPCR Premix (Takara). GAPDH was selected as the internal reference for the expression of mRNA. Besides, the gene expression of miRNA was normalized to U6 as an endogenous control. Normalizing gene expression was calculated by means of the 2^−ΔΔCT^ method. Each test was duplicated at least three times. The synthesized sequences of primers are listed in Additional file 11: Table S3.

**Western blotting**

Total proteins of OS cell lines (or OS tissues) were obtained by using radioimmunoprecipitation assay (RIPA) plus phenylmethylsulfonyl fluoride (PMSF) at a ratio of 100:1 following the protocol (both from Beijing Solarbio Science & Technology Co., Ltd., Beijing, China). In addition, protein levels were measured by bicinchoninic acid assay (BCA) (cat. no. PC0020; Beijing Solarbio Science & Technology Co., Ltd.). The different molecular weight of proteins was isolated by SDS-PAGE and then shifted to PVDF membranes (Millipore, USA). At room temperature, these membranes were blocked with 7% skim milk (BD, Difco™) for one hour, after which they were incubated with specialized primary antibodies (all 1:1000) at 4°C overnight and then washed at least four times with TBST for five minutes each time. Ultimately, these membranes were hatched with secondary antibody (1:2000; IgG Rabbits) for 90 minutes at room temperature and were then washed at least four times with TBST for five minutes each time. GAPDH is used as an internal reference for measuring the level of protein. The protein signal was detected by Tanon 5200 automatic chemiluminescence imaging analysis system (Tanon Science & Technology Co., Ltd, Shanghai, China) *via* ECL Western blotting Substrate (cat. no. PE0010; Beijing Solarbio Science & Technology Co., Ltd.). Moreover, the intensity of proteins was weighed by ImageJ software (National Institutes of Health, Bethesda, MD, USA). Each test was duplicated at least three times.

**CCK-8 proliferation assay**

Cell proliferation was measured by Cell Counting Kit-8 (CCK-8, Beyotime, Shanghai, China). Birefly, these OS cell lines (3.5×10^3^ cells) that were stably infected with Nectin-4-OE, Vector-NC, shNectin-4, and shCtrl lentivirus were put in 96-well plates and cultured for 0, 12, 24, 36, 48 and 72h. At each time point, CCK-8 reagent was added to each well and incubated for another 1 h at 37°C. The absorbance of each well at a wavelength of 450 nm was recorded from a microplate reader, after which we drew the cell proliferation curves. Each test was duplicated for at least three times.

**Colony formation assay**

The 1×10^3^ OS cells with stable clones were plated in a 6-well plate and cultured for 1~2 weeks. After being washed with phosphate-buffered saline (PBS) three times, the surviving cell clusters were fixed with 4% paraformaldehyde (Sigma-Aldrich Co., St. Louis, MO USA) for half an hour and then stained with 0.01% crystal violet for a quarter of an hour. All experiments were carried out in triplicate.

**Migration and invasion assay**

Cell migration and invasion capacity were detected by transwell assays *via* chamber inserts (BD Biosciences) covered with Matrigel (invasion capacity detection) or without Matrigel (migration capacity detection). The operation consisted of the following steps: the stably infected 143B, MG63 (both, 3×10^4^), and U2OS cells (2.5×10^4^) were seeded into the upper chamber and re-suspended with 200μl of DMEM medium containing 0.1% BSA without FBS. Meanwhile, 500ul of the complete medium was put into the lower chamber as a driving factor. The cells were used for testing migration and invasion ability after being cultured for 24 h and 48h, respectively. Finally, the bottom of these transwell chambers was washed by PBS. After drying, those cells were stained with 0.1% crystal violet for 10 min. The migrated cells were observed by an inverted microscope (Olympus cellSens Entry 1.16; Olympus Corporation).

**Wound healing assay**

The OS cells lines (1×10^6^) from Nectin-OE, Vector-NC, shNectin-4, and shCtrl were cultured in 6-well plates. After the cell reached 90 % confluence, a line was drawn using a marker on the bottom of the dish, and then a sterile 10μl pipet tip was used to scratch three separate wounds through the cells, moving perpendicular to the line. The cells were gently rinsed twice with PBS to remove floating cells and incubated in a serum-free medium in a CO_2_ incubator. Images of the scratches were taken using an inverted microscope (Olympus cellSens Entry 1.16; Olympus Corporation) at 0 and 24 h of incubation. The comparison among wound widths was calculated by relative migration distance.

**Enrichment Analysis**

The PID_PI3KCI_PATHWAY gene set was downloaded from the MsigDB database. The gene set variation analysis (GSVA) was performed to study the underlying gene sets with expression changes on osteosarcoma samples from the TARGET database. Subsequently, the difference in GSVA score between the high Nectin-4 expression group and low expression group was analyzed to detect whether there was a difference in PI3K pathway activity between the two groups. In addition, gene set enrichment analysis (GSEA) was also used to explore the relationship between PI3K pathway enrichment and Nectin-4 expression by using software GSEA V2.2.0 (downloaded from http://www.gsea-msigdb.org/gsea/msigdb/index.jsp).

**Luciferase reporter assay**

The reporter plasmids (pmirGLO-Firefly_Luciferase-Renilla_Luciferase) containing the ATK1 or P65 3’UTR wild type (Wt) sequence or a mutant type (Mt) sequence targeted and bonded to miR-520c-3p were designed by ^®^Sangon Biotech (Shanghai, China). 293T cells were co-transfected with the reporter plasmid and miR-520c-3p mimic or inhibitor, after which they were incubated for 24 h. Furthermore, the effect of miR-520c-3p on the luciferase reporter with the AKT1 3'-UTR (Wt vs Mt) and P65 3'-UTR (Wt vs. Mt) was evaluated by relative firefly luciferase activity (normalized to Renilla luciferase activity).
