# Supplementary material for: Nectin-4 promotes osteosarcoma progression and metastasis through activating PI3K/AKT/NF-κB signaling by down-regulation of miR-520c-3p
Source: Cancer Cell Int. 2022 Aug 11;22:252. doi: 10.1186/s12935-022-02669-w (PMC9367085; doi:10.1186/s12935-022-02669-w)
Supplement: Supplementary file 9 — Additional file 9: Table S1. Potential mircoRNAs targeting both AKT1 and P65 were predicted by six online bioinformatics analysis software databases by using Venn diagrams. [file 12935_2022_2669_MOESM9_ESM.docx]

Additional file 9: Table S1. Potential mircoRNAs targeting both AKT1 and P65 were predicted by six online bioinformatics analysis software databases by using Venn diagrams.

| Both AKT1 AND P65 | AKT1 (databases) | P65 (databases) |
| --- | --- | --- |
| miR-22-3p | PITA | microT |
| miR-138-5p | PITA/miRmap/miRanda | miRmap/microT/miRanda/PicTar/TargetScan |
| miR-143-3p | PITA | miRanda |
| miR-185-5p | PITA/miRanda | miRmap/microT |
| miR-302a-3p | PITA | RNA22/miRmap/microT/miRanda |
| miR-296-5p | PITA/miRmap | miRmap |
| miR-302b-3p | PITA/miRmap/miRanda/TargetScan | RNA22/miRmap/microT/miRanda |
| miR-302c-3p | PITA/miRmap/miRanda/TargetScan | RNA22/miRmap/microT/miRanda |
| miR-302d-3p | PITA/miRmap/miRanda/TargetScan | RNA22/miRmap/microT/miRanda |
| miR-372-3p | PITA/miRmap/miRanda/TargetScan | RNA22/miRmap/microT/miRanda |
| miR-373-3p | PITA/miRmap/miRanda/TargetScan | RNA22/miRmap/microT/miRanda |
| miR-512-3p | PITA | microT |
| miR-520e | PITA/miRmap/miRanda/TargetScan | miRmap/microT/miRanda/TargetScan |
| miR-520f-3p | PITA | miRmap/microT |
| miR-520a-3p | PITA/miRmap/miRanda/TargetScan | miRmap/microT/miRanda |
| miR-520b | PITA/miRmap/miRanda/TargetScan | miRmap/microT/miRanda |
| miR-520c-3p | PITA/miRmap/miRanda/TargetScan | miRmap/microT/miRanda/TargetScan |
| miR-520d-3p | PITA/miRmap/miRanda/TargetScan | miRmap/microT/miRanda/TargetScan |
| miR-654-5p | PITA | RNA22 |
| miR-361-3p | PITA/miRmap | miRmap |
| miR-541-3p | PITA | miRmap |
| miR-744-5p | PITA | RNA22 |
| miR-1270 | PITA | miRmap |
| miR-302e | PITA/miRmap/miRanda/TargetScan | miRmap/microT/miRanda |
| miR-378g | miRmap | RNA22 |
| miR-4731-5p | RNA22 | miRmap |
| miR-4739 | miRmap | miRmap |
| miR-1306-5p | RNA22 | miRmap/microT |
