# Supplementary material for: Nectin-4 promotes osteosarcoma progression and metastasis through activating PI3K/AKT/NF-κB signaling by down-regulation of miR-520c-3p
Source: Cancer Cell Int. 2022 Aug 11;22:252. doi: 10.1186/s12935-022-02669-w (PMC9367085; doi:10.1186/s12935-022-02669-w)
Supplement: Supplementary file 10 — Additional file 10: Table S2. Association between Nectin-4 expression and clinicopathologic features in TARGET [file 12935_2022_2669_MOESM10_ESM.docx]

**Additional file 10: Table S2.** Association between Nectin-4 expression and clinicopathologic features in TARGET

| Variable |  | No. | Nectin-4 expression | | х^2^ | P-value |
| --- | --- | --- | --- | --- | --- | --- |
|  |  |  | High | Low |  |  |
| Gender |  |  |  |  | 0.104 | 0.828 |
|  | Male | 50 | 22(25.29%) | 28(32.18%) |  |  |
|  | Female | 37 | 15(17.24%) | 22(25.29%) |  |  |
| Age |  |  |  |  | 0.06 | 0.831 |
|  | ≥15Y | 41 | 18(20.69%) | 23(26.44%) |  |  |
|  | <15Y | 46 | 19(21.84%) | 27(31.03%) |  |  |
| Race |  |  |  |  | 3.743 | 0.154 |
|  | White | 52 | 18(27.27%) | 35(53.03%) |  |  |
|  | Black | 7 | 5(7.6%) | 2(3.03%) |  |  |
|  | Asian | 7 | 2(3.03%) | 5(7.6%) |  |  |
| Metastasis |  |  |  |  | 0.032 | 0.859 |
|  | Yes | 22 | 9(10.34%) | 13(14.94%) |  |  |
|  | No | 65 | 28(32.18%) | 37(42.53%) |  |  |
| Metastasis site |  |  |  |  | 0.151 | 0.697 |
|  | Lung | 17 | 8(34.78%) | 9(39.13%) |  |  |
|  | Bone | 1 | 0(0.00%) | 1(4.35%) |  |  |
|  | Both | 5 | 2(8.7%) | 3(13.04%) |  |  |
| Primary tumor site |  |  |  |  | 0.858 | 0.354 |
|  | Leg/Foot | 81 | 35(40.23%) | 46(52.87%) |  |  |
|  | Arm/Hand | 6 | 2(2.30%) | 4(4.60%) |  |  |
| Specific tumor region |  |  |  |  | 0.245 | 0.620 |
|  | Distal | 28 | 13(24.07%) | 15(27.78%) |  |  |
|  | Proximal | 23 | 10(18.52%) | 13(24.04%) |  |  |
|  | Posterior | 3 | 1(1.85%) | 1(1.85%) |  |  |
| Primary site progression |  |  |  |  | 1.654 | 0.437 |
|  | Yes | 15 | 7(39.47%) | 8(21.05%) |  |  |
|  | No | 23 | 12(31.58%) | 11(28.95%) |  |  |
| Time to first relapse |  |  |  |  | 0.173 | 0.677 |
|  | ≥19.5 | 11 | 6(15.79%) | 5(13.16%) |  |  |
|  | <19.5 | 27 | 11(28.95%) | 16(42.11%) |  |  |
| Histologic Stage |  |  |  |  | 3.520 | 0.172 |
|  | 1-2 | 18 | 11(32.35%) | 7(20.59%) |  |  |
|  | 3-4 | 16 | 7(20.59%) | 9(26.47%) |  |  |

Using Chi-square test or Fisher’s exact test
