# Supplementary material for: Nectin-4 promotes osteosarcoma progression and metastasis through activating PI3K/AKT/NF-κB signaling by down-regulation of miR-520c-3p
Source: Cancer Cell Int. 2022 Aug 11;22:252. doi: 10.1186/s12935-022-02669-w (PMC9367085; doi:10.1186/s12935-022-02669-w)
Supplement: Supplementary file 11 — Additional file 11: Table S3. Primer sequences for quantitative real-time PCR and shRNA directing at the human Nectin-4 sequence [file 12935_2022_2669_MOESM11_ESM.docx]

**Additional file 11: Table S3**. Primer sequences for quantitative real-time PCR and shRNA directing at the human Nectin-4 sequence

| **Gene** | **Sequences** | **Sequences** |
| --- | --- | --- |
| **mRNA/miRNA** | **Forward primer** | **Backward primer** |
| Nectin-4  Vimentin  N-Cadherin  Slug  Zeb1  Zo-1  GAPDH­  miR-302d-3p  miR-520d-3p  miR-302c-3p  miR-302b-3p  miR-520c-3p  miR-520a-3p  miR-520b  miR-302e  U6  **shRNA**  ShNectin-4#1  ShNectin-4#2  ShNectin-4#3  ShCtrl | 5′-CCCAAGCTTATGCCCCTGTCCCTGGGAGCCGA-3′  5′-CGCTCCTACGATTCACAGCC-3′  5′-GAGATCCTACTGGACGGTTCG-3′  5′-TGCGGCAAGGCGTTCCAGA-3′  5′-GCACAACCAAGTGCAGAAGA-3′  5′-TGGCCACAGCCCGAGGCATA-3′  5′-TGACTTCAACAGCGACACCCA-3′  5′- TGCTTCCATGTTTGAGTGTG-3′  5′-GGTCTACAAAGGGAAGC-3′  5′-GCGTGCTTCCATGTTTCAGTGG-3′  5′- GCGTAAGTGCTTCCATGTT -3′  5′-GCCGCCAAAGTGCTTCCTTTTAG-3′  5′- ACACTCCAGCTGGGAAAGTGCTTCCC-3′  5′-AAGTGCTTCCTTTTAGAGG-3′  5′-CTCATCGCATAAGTGCTTCCAT-3′  5′-CTCGCTTCGGCAGCACATATACTA-3′  **Sequences**  5′-CAGAGCAGTATTAATGATGCA-3′  5′-CACTCCAAATACGGGCTTCAT-3′  5′-CTGGTCCAGCACTAGAAGA-3′  5′-TTCTCCGAACGTGTCACGT-3′ | 5′-CTAGTCTAGAGACCAGGTGTCCCCGCCCATTGA-3′  5′-TGTGGACGTGGTCACATAGC-3′  5′-TCTTGGCGAATGATCTTAGGA-3′  5′-CAGTGTGCTACACAGCAGCCAGA-3′  5′-CATTTGCAGATTGAGGCTGA-3′  5′-GTAAGCGCAGCTCCACAGGC-3′  5′-CACCCTGTTGCTGTAGCCAAA-3′  5′-GAACATGTCTGCGTATCTC-3′  5′-TTTGGCACTAGCACATT-3′  5′- CAGTGCAGGGTCCGAGGTAT-3′  5′-TCCAGGGACCGAGGA-3′  5′-TCGCACTGGATACGACACCCTC-3′  5′-CTCAACTGGTGTCGTGGA-3′  5′-GAACATGTCTGCGTATCTC-3′  5′-TATCGTTGTTCTCGACTCCTTCAC-3′  5′-CGAATTTGCGTGTCATCCTTGCG-3′ |
